# Trends in gestational age at live birth in Scotland from 2005 to 2019: a population-based study

**DOI:** 10.12688/wellcomeopenres.20916.1

**Published:** 2024-05-15

**Authors:** Emily Moore, Sonya Scott, Jeeva John, Clara Calvert, Rachael Wood, Sarah J. Stock

**Affiliations:** 1 Public Health Scotland, Edinburgh, EH12 9EB, UK; 2 Population Health Sciences, University of Southampton, Southampton, England, SO17 1BJ, UK; 3 Usher Institute, The University of Edinburgh, Edinburgh, Scotland, EH16 4UX, UK; 4 MRC Centre for Reproductive Health, The University of Edinburgh, Edinburgh, Scotland, EH16 4UU, UK

**Keywords:** Pregnancy, Premature birth, Term birth, Post-term birth, Epidemiology, Maternal age, Social deprivation, Scotland

## Abstract

**Background:**

Gestation at birth is associated with short and long-term outcomes. This study used high quality, national, administrative data to examine trends in gestation at birth in Scotland.

**Methods:**

This observational study used maternity hospital discharge records for 2005 to 2019 to determine trends in the percentage of live births that were preterm (<37 weeks gestation), term (37–41 weeks), and post-term (≥42 weeks), overall and by maternal age and deprivation category. Preterm births were further examined by category of preterm birth (moderate to late [32–36 weeks]; very [28–31 weeks]; extremely [<28 weeks] preterm), and onset of labour (spontaneous; provider-initiated). Singleton and multiple births were examined separately. Aggregate logistic regression was used to estimate the annual change in the odds of a birth being in a specified gestational category.

**Results:**

The percentage of singleton births in Scotland that were preterm decreased from 2005 (6.5%, 3,361/51,665) to 2010 (5.8%, 3268/56344), then increased to 2019 (7.2%, 3,408/47,507). The percentage of singleton births that were spontaneous moderate to late, very, and extremely preterm all increased between 2010 and 2019. The percentage of singleton births that were provider-initiated moderate to late preterm also increased between 2010 and 2019, however provider-initiated very or extremely preterm birth decreased. The percentage of singleton births that were preterm increased over time across all maternal age and deprivation categories, with increases greatest in groups at highest baseline risk. The percentage of singleton births that were post-term increased from 2005 to 2009, then decreased to 2019.

**Conclusions:**

There has been an increase in spontaneous preterm birth from 2010 to 2019, which is not fully explained by changes in maternal age or deprivation. Further research to examine the contribution of other, preventable, risk factors is warranted. Trends in provider-initiated preterm birth, and post-term birth, are likely to reflect changing clinical practice.

## Plain language summary

Most babies are born between 37 and 42 weeks gestation. Some babies are born earlier (preterm) birth, or later (post-term) birth, and either can lead to health complications for babies.

For preterm birth, the earlier a baby is born, the higher the chance of problems, such as lung disease or cerebral palsy. Preterm birth can either occur after spontaneous onset of labour (spontaneous preterm birth) or result from artificially starting labour (induction of labour) or caesarean birth before labour (provider-initiated preterm birth). Provider-initiated birth is offered when the risks of a pregnancy continuing are considered higher than the risks of the baby being born, for example, when the risk of stillbirth is high. Women whose pregnancy continues past their due date may be offered induction of labour to avoid complications associated with post-term birth such as stillbirth.

Women who are older or who live in deprived areas have higher rates of preterm birth and stillbirth than younger mothers who live in affluent areas. Changes in the social and demographic makeup of the pregnant population can thus influence preterm and post-term birth rates. Changes in maternity care (e.g. guidance on induction of labour) can also influence the chance of provider-initiated preterm and post-term birth.

We used maternity data from all hospitals in Scotland for 2005 to 2019 to understand trends in preterm and post-term births over time. We found an overall increase in spontaneous preterm birth from 2010 to 2019. Provider-initiated moderate to late preterm births at 32–36 weeks gestation increased, but provider-initiated births before 32 weeks gestation decreased. These trends were not fully explained by changes in mothers’ age or socioeconomic status. We also found that post-term birth decreased between 2009 and 2019. Further research to understand the reasons underlying these trends is needed.

## Introduction

Gestational age at birth is associated with adverse health outcomes for offspring, with increased risk following preterm (<37 weeks gestation) and post-term (≥42 weeks gestation) birth ^1– 6^. Globally it is estimated that 15 million (10.6%) of babies are born preterm each year, with regional percentages ranging from 8.7% of live births in Europe to 13.4% in North Africa ^7^. Preterm birth is the leading cause of neonatal mortality ^2^, and preterm infants are at greater risk of short and long-term health problems ^1^. The economic impact of preterm birth is substantial, with an estimated cost of $26.2 billion per year in the United States alone ^8^.

A study published in 2014 reported absolute increases in the percentage of births that were preterm between 1990 and 2014 in high income countries ^7^. Potential reasons for this upward trend include those related to changes in: data quality; clinical practice (e.g., ability to support earlier foetal viability and/or increase in provider-initiated preterm birth to prevent foetal death); increasing maternal comorbidity (e.g., increased prevalence of maternal obesity and diabetes); and sociodemographic factors (e.g., older maternal age, increasing multiple births, worsening deprivation) ^7, 9– 13^. This is supported by the findings in some studies of varying trends by onset of labour (i.e. spontaneous [birth preceded by preterm pre-labour rupture of membranes or spontaneous labour] or provider-initiated [induction of labour or pre-labour caesarean section]) and by category of preterm birth (moderate to late, very, and extremely preterm) ^14– 17^. It has been acknowledged, however, that more work is required to address the limitations of current literature, in particular with respect to clear and consistent inclusion criteria for gestational age categories, reliable gestational age ascertainment, reporting of trends for singleton and multiple births separately, and for preterm birth sub-types, categorised by category of preterm birth and onset of labour ^7, 13, 18^.

Whilst there is accumulating evidence of risks associated with post-term gestation ^3– 6^, there are no recent published analyses of longer-term national trends in the percentage of births that are post-term. In Europe, post-term births generally account for <1% of all births, but there is substantial variation across countries with percentages ranging from 0.1% to 6% of live births ^19^.

The aim of this paper is therefore to describe recent trends in gestational age at birth in Scotland, a high-income country with one of the highest levels of preterm birth in Europe ^19^. Using high quality, population-based administrative data we describe long-term trends in gestational age at live birth for singleton and multiple births separately, overall and by maternal age and deprivation category. For preterm births, we further describe trends by category of preterm birth and onset of labour (spontaneous or provider-initiated).

## Methods

### Data source and quality

We analysed data from the Scottish Morbidity Record 2 (SMR02) database held by Public Health Scotland (PHS). An SMR02 record is returned to PHS from hospitals across Scotland when a woman is discharged from an episode of day case or in-patient care in a maternity unit or following an attended home birth (≤2% of births in Scotland) ^20^. The SMR02 database is highly complete for live births in Scotland, including at least 98% of all statutorily registered live births ^12^. SMR02 records returned following an episode of care involving a birth include additional information ^21^, including gestation at birth in completed weeks. Recording of gestation on SMR02 is highly complete and accurate when compared against the best obstetric estimate noted in the clinical record ^22^: gestation at birth was available on 99.8% of SMR02 live birth records over our study period.

Whether the onset of labour was spontaneous [birth preceded by preterm pre-labour rupture of membranes or spontaneous labour] or provider-initiated [induction of labour or pre-labour caesarean section] is appended to SMR02 records for preterm births by PHS according to an internally developed algorithm. This draws on ICD-10 diagnostic codes plus variables recording induction of labour, duration of labour, and mode of delivery as shown in Box 1. This algorithm assigned onset of labour status to >99.9% of SMR02 live preterm birth records over our study period. Completeness of other key variables was also very high. Maternal age and deprivation category (SIMD quintile, see below) were available on >99.9% and 99.7% of SMR02 live birth records including gestation over our study period.

**Box 1. Summary of algorithm used to assign onset of labour status (spontaneous or provider-initiated) to live preterm births**

**Table:** 

Group	Diagnostic code for pre-labour rupture of membranes *	Induction of labour	Mode of delivery	Duration of labour	Onset of labour category assigned
1	Yes	Any	Any	Any	Spontaneous (with P-PROM)
2	No	Yes	Any	Any	Provider-initiated (IoL)
3	No	No or unknown	Any vaginal	Any	Spontaneous (without P-PROM)
4	No	No or unknown	Any CS	0	Provider-initiated (Pre-labour CS)
5	No	No or unknown	Any CS	>0	Spontaneous (without P-PROM)
6	No	No or unknown	Elective CS	Unknown	Provider-initiated (Pre-labour CS)
7	No	No or unknown	Emergency CS	Unknown	Spontaneous (without P-PROM)

* ICD10 code O42.0 – O42.9 recorded P-PROM is preterm pre-labour rupture of membranes; IoL is induction of labour; CS is caesarean section The algorithm is applied hierarchically using data available on SMR02 birth records, i.e. in step 1, all preterm births are assessed to identify those in group 1; in step 2, the remaining births are assessed to identify those in group 2; and so on

### Setting and participants

For this study, the PHS Pregnancy, Births, and Child Health analyst team provided the research team with fully anonymised, aggregate data files providing counts of live births by gestational age, year of birth, and (separately) maternal age, deprivation level, and (for preterm births) onset of labour (data extracted on 03/10/2022). Data was provided for singleton and multiple live births separately. Births following a multiple pregnancy (of twins or more) were included if one or more baby was live born, and were counted as one birth.

Data was provided on all live births between 22 weeks and 0 days (22 ^+0^) and 44 ^+6^ weeks gestation in Scotland, between 1 ^st^ January 2005 and 31 ^st^ December 2019. The start date of 2005 was chosen as a previous study of preterm birth reported trends to 2004 ^23^. Our original intention was to include the most recent data available at commencement of the study, however, due to potential effects of pandemic mitigation measures on the risk of preterm birth ^24^, we censored analysis at 2019.

### Statistical analysis

Analyses followed a pre-specified protocol (available at: https://github.com/Public-Health-Scotland/preterm_trends_public ^25^). We examined counts of live births by single gestational week (22 to 44 completed weeks inclusive), and gestational age category, and year of birth. Gestational age was categorised according to established international definitions ^26^ as preterm (<37 weeks gestation); term (37–41 weeks gestation) and post term (≥42 weeks gestation). We then examined counts of live preterm births sub-categorised into moderate to late preterm [32–36 weeks], very preterm [28–31 weeks], and extremely preterm [<28 weeks], and onset of labour (spontaneous or provider-initiated).

Counts of live births by gestational age categories (preterm, term, and post-term), year of birth, and, separately, maternal age and deprivation categories were then examined. Maternal age was categorised as <20 years, 20–24 years, 25–29 years, 30–34 years, 35–39 years and ≥40 years. Maternal deprivation was categorised using the Scottish Index of Multiple Deprivation (SIMD) ^27, 28^. The SIMD ranks small areas across Scotland by level of material deprivation based on administrative data relating to income, employment, education, health, access to services, crime, and housing. Areas are then categorised into five ordered quintiles (with SIMD quintile 1 reflecting the highest level of deprivation, and SIMD quintile 5 the lowest), each including approximately 20% of the Scottish population. Women were allocated to ranked area, and hence SIMD quintile, based on their postcode of residence at end of pregnancy.

We plotted the percentage of all live births occurring in each single gestational week (22 to 44 completed weeks inclusive) in four evenly spaced years (2005, 2010, 2015 and 2019) to visually assess any change in gestational distribution at birth during the study period.

We calculated the percentage of all live births that were preterm, term, and post-term in each year. We also calculated the percentage of all live births that were in the preterm birth sub-categories (e.g., extremely preterm, spontaneous onset) in each year. Finally, we calculated the percentage of all live births to women in specified maternal age and, separately, deprivation categories that were preterm, term, and post-term in each year. As the completeness of relevant variables was so high on the source SMR02 records, all percentages were based on the number of babies with known gestation and, where applicable, also maternal age or SIMD quintile.

We calculated the absolute and relative difference in the percentage of births that were preterm, term, and post-term in 2019 compared to 2005. The absolute difference was calculated as the percentage in 2019 minus the percentage in 2005. The relative difference was calculated by dividing the absolute difference by the baseline percentage in 2005 and multiplying by 100. Aggregate, univariate, logistic regression weighted for the number of live births in each year was used to estimate the average change per year in the unadjusted odds of a birth being preterm, term and post-term.

All analyses were performed for live singleton and multiple births separately.

R version 4.1.2 was used to conduct all statistical analyses. Two analysts (EM and CC) independently checked data and results. Analysis code and a completed STROBE reporting checklist for this study are publicly available ( https://github.com/Public-Health-Scotland/preterm_trends_public ^25^).

### Ethics and data availability

All analyses were undertaken within Public Health Scotland on anonymised, aggregate data following established organisational procedures. No additional ethical or information governance approvals were required. Aggregate data from SMR02 is available through the Scottish Health and Social Care Open Data Platform ^29^. Bespoke research extracts of pseudonymised, patient level data can be provided through the Scottish national safe haven facility, subject to relevant approvals ^30^.

## Results

### Singleton births

There were 816,058 live births in Scotland between 2005 and 2019. Of these 803,842 (98.5%) were singleton births, of which 802,217 (99.8%) had known gestational age at birth. The annual number of singleton births in Scotland initially increased from 51,685 in 2005 to 57,197 in 2008, then gradually declined to 47,519 in 2019 ( Table 1).

**Table 1.** Number (%) of births that were preterm, term, and post-term, Scotland 2005–2019. (a) Live singleton births

Singleton births		Total 2005–2019	Year															Absolute difference 2019 v 2005	Relative (%) difference 2019 v 2005
			2005	2006	2007	2008	2009	2010	2011	2012	2013	2014	2015	2016	2017	2018	2019		
**Total singleton** ** births**	Number	803842	51685	52709	55558	57197	56383	56377	56296	55625	53820	54596	53022	52624	51025	49406	47519	-4166	-8.1%
**Unknown** ** gestation**	Number	1625	20	33	49	56	50	33	36	63	73	246	239	414	272	29	12	-8	-40.0%
**Total singleton** ** births with** ** known** ** gestation**	Number	802217	51665	52676	55509	57141	56333	56344	56260	55562	53747	54350	52783	52210	50753	49377	47507	-4158	-8.0%
**Preterm**	Number	50946	3361	3311	3409	3669	3476	3268	3322	3296	3235	3305	3546	3463	3481	3396	3408	47	1.4%
	%	6.4	6.5	6.3	6.1	6.4	6.2	5.8	5.9	5.9	6.0	6.1	6.7	6.6	6.9	6.9	7.2	0.7	10.3%
**Term**	Number	732073	46863	47832	50491	51673	51088	51526	51558	50894	49335	49913	48206	47829	46341	45190	43334	-3529	-7.5%
	%	91.3	90.7	90.8	91.0	90.4	90.7	91.4	91.6	91.6	91.8	91.8	91.3	91.6	91.3	91.5	91.2	0.5	0.6%
**Post-** **term**	Number	19198	1441	1533	1609	1799	1769	1550	1380	1372	1177	1132	1031	918	931	791	765	-676	-46.9%
	%	2.4	2.8	2.9	2.9	3.1	3.1	2.8	2.5	2.5	2.2	2.1	2.0	1.8	1.8	1.6	1.6	-1.2	-42.3%

**Table:** (b) Live multiple births

Multiple births		Total 2005–2019	Year															Absolute difference 2019 v 2005	Relative (%) difference 2019 v 2005
			2005	2006	2007	2008	2009	2010	2011	2012	2013	2014	2015	2016	2017	2018	2019		
**Total multiple ** **births**	Number	12216	769	805	867	929	900	905	827	836	779	837	790	790	745	741	696	-73	-9.5%
**Unknown ** **gestation**	Number	18	1	0	0	0	1	0	0	0	0	3	3	7	3	0	0	-1	-
**Total multiple ** **births with ** **known** ** gestation**	Number	12198	768	805	867	929	899	905	827	836	779	834	787	783	742	741	696	-72	-9.4%
**Preterm**	Number	7158	442	431	458	506	453	490	465	468	472	502	509	530	507	483	442	0	0.0%
	%	58.7	57.6	53.5	52.8	54.5	50.4	54.1	56.2	56.0	60.6	60.2	64.7	67.7	68.3	65.2	63.5	6.0	10.3%
**Term**	Number	5038	326	373	408	423	446	415	362	368	307	332	278	253	235	258	254	-72	-22.1%
	%	41.3	42.4	46.3	47.1	45.5	49.6	45.9	43.8	44.0	39.4	39.8	35.3	32.3	31.7	34.8	36.5	-6.0	-14.0%
**Post-term**	Number	2	0	1	1	0	0	0	0	0	0	0	0	0	0	0	0	-	-
	%	0.0	0.0	0.1	0.1	0.0	0.0	0.0	0.0	0.0	0.0	0.0	0.0	0.0	0.0	0.0	0.0	-	-

Gestational age categories are: preterm <37 weeks; term 37–41 weeks; post term ≥42 weeks % based on births with known gestation

***Gestation at birth.*** Over the total study period, 50,946 (6.4%) of all singleton births were preterm; 732,073 (91.3%) were born at term; and 19,198 (2.4%) were post-term ( Table 1).

**Figure 1.**
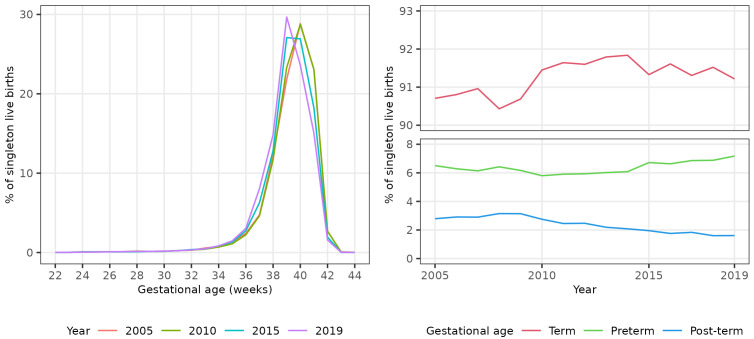
Trends in gestational age at birth, singleton live births, Scotland. **Figure 1a** shows the distribution of gestational age at birth for live singleton births in Scotland in 2005, 2010, 2015 and 2019. **Figure 1b** shows trends in the percentage of all live singleton births in Scotland between 2005 and 2019 that were preterm, term, and post-term.

There was a shift in the distribution of singleton births between 2005 and 2019 to earlier gestational ages ( Figure 1), with the percentage of all singleton births occurring at 40 ^+0^ weeks gestation or later declining from 54.7% (28,256 / 51,665) in 2005 to 40.2% (19,115 / 47,507) in 2019 ( Table 2). The percentage of all singleton births that were pre- and post-term showed non-linear trends over the study period. The percentage that were preterm initially decreased until 2010, then increased to 2019. Conversely, the percentage that were post-term initially increased until 2009, then decreased to 2019 ( Figure 1).

**Table 2.** Number (%) of births by week of gestation, Scotland, selected years. (a) Live singleton births

Singleton births Year		Week of gestation																						
		22	23	24	25	26	27	28	29	30	31	32	33	34	35	36	37	38	39	40	41	42	43	44
**2005**	Number	4	9	47	41	50	55	93	74	93	127	151	291	393	689	1244	2453	6314	11281	14906	11909	1385	44	12
	%	0.0	0.0	0.1	0.1	0.1	0.1	0.2	0.1	0.2	0.2	0.3	0.6	0.8	1.3	2.4	4.7	12.2	21.8	28.9	23.1	2.7	0.1	0.0
**2010**	Number	6	15	36	43	56	62	85	76	85	130	172	234	388	618	1262	2606	6590	13137	16201	12992	1522	22	6
	%	0.0	0.0	0.1	0.1	0.1	0.1	0.2	0.1	0.2	0.2	0.3	0.4	0.7	1.1	2.2	4.6	11.7	23.3	28.8	23.1	2.7	0.0	0.0
**2015**	Number	6	11	40	44	43	48	49	73	82	124	195	243	441	700	1447	3312	6771	14296	14210	9617	1022	7	2
	%	0.0	0.0	0.1	0.1	0.1	0.1	0.1	0.1	0.2	0.2	0.4	0.5	0.8	1.3	2.7	6.3	12.8	27.1	26.9	18.2	1.9	0.0	0.0
**2019**	Number	6	14	17	28	46	55	64	69	83	109	152	225	394	697	1449	3826	7058	14100	11215	7135	756	8	1
	%	0.0	0.0	0.0	0.1	0.1	0.1	0.1	0.1	0.2	0.2	0.3	0.5	0.8	1.5	3.1	8.1	14.9	29.7	23.6	15.0	1.6	0.0	0.0

**Table:** (b) Live multiple births

Multiple births Year		Week of gestation																						
		22	23	24	25	26	27	28	29	30	31	32	33	34	35	36	37	38	39	40	41	42	43	44
**2005**	Number	1	1	4	3	7	12	8	14	20	25	33	43	65	91	115	153	142	27	4	0	0	0	0
	%	0.1	0.1	0.5	0.4	0.9	1.6	1.0	1.8	2.6	3.3	4.3	5.6	8.5	11.8	15.0	19.9	18.5	3.5	0.5	0.0	0.0	0.0	0.0
**2010**	Number	0	0	8	3	3	9	9	17	26	23	51	39	62	84	156	203	189	18	5	0	0	0	0
	%	0.0	0.0	0.9	0.3	0.3	1.0	1.0	1.9	2.9	2.5	5.6	4.3	6.9	9.3	17.2	22.4	20.9	2.0	0.6	0.0	0.0	0.0	0.0
**2015**	Number	0	2	2	2	5	6	8	13	14	14	37	44	67	100	195	229	42	3	4	0	0	0	0
	%	0.0	0.3	0.3	0.3	0.6	0.8	1.0	1.7	1.8	1.8	4.7	5.6	8.5	12.7	24.8	29.1	5.3	0.4	0.5	0.0	0.0	0.0	0.0
**2019**	Number	1	0	2	1	6	4	12	8	11	19	21	29	73	96	159	240	11	3	0	0	0	0	0
	%	0.1	0.0	0.3	0.1	0.9	0.6	1.7	1.1	1.6	2.7	3.0	4.2	10.5	13.8	22.8	34.5	1.6	0.4	0.0	0.0	0.0	0.0	0.0

% based on births with known gestation

The percentage of all singleton births that were preterm increased from 6.5% in 2005 to 7.2% in 2019, an absolute increase of 0.7% and a relative increase of 10.3%. The unadjusted odds of a singleton birth being preterm increased on average by 1% per year between 2005 and 2019 (OR 1.01, 95% CI 1.01 - 1.01). Conversely, the percentage of all singleton births that were post-term decreased from 2.8% in 2005 to 1.6% in 2019, an absolute decrease of 1.2% and a relative decrease of 42.3%. The unadjusted odds of a singleton birth being post-term decreased on average by 5% per year (OR 0.95, 95% CI 0.95 – 0.95). The increase in the percentage of singleton births that were preterm was more than offset by the decrease in the percentage that were post-term: the percentage of singleton births that were at term therefore increased slightly between 2005 and 2019 (OR 1.01, 95% CI 1.01 - 1.01) ( Table 3).

**Table 3.** Percentage of births that were preterm, term, and post-term, Scotland 2005–2019. (a) Live singleton births

Singleton births	% of all live singleton births in stated category, 2005	% of all live singleton births in stated category, 2019	Absolute difference 2019 v 2005 (95% CI)	Relative (%) difference 2019 v 2005 (95% CI) *	Unadjusted Odds ratio (95% CI)
**All Preterm**	6.5 (6.3 - 6.7)	7.2 (7.2 - 7.2)	0.7 (0.4 - 1.0)	10.3% (1.5 - 16.3)	1.01 (1.01 - 1.01)
*Spontaneous*	3.9 (3.4 - 4.3)	4.4 (3.9 - 4.8)	0.5 (0.2 - 0.7)	12.7% (4.4 - 18.9)	1.01 (1.00 - 1.01)
*Provider-initiated*	2.6 (2.2 - 3.1)	2.8 (2.3 - 3.3)	0.2 (0.0 - 0.4)	6.2% (2.7 - 9.0)	1.01 (1.01 - 1.01)
**Moderate to late preterm**	5.4 (5.1 - 5.6	6.1 (5.9 - 6.4)	0.8 (0.5 - 1.1)	14.6 (3.5 - 22.6)	1.01 (1.01 - 1.01)
*Spontaneous*	3.2 (2.8 - 3.7)	3.6 (3.2 - 4.1)	0.4 (0.2 - 0.6)	12.1% (4.9 - 17.6)	1.01 (1.00 - 1.01)
*Provider-initiated*	2.1 (1.7 - 2.6)	2.5 (2.0 - 3.0)	0.4 (0.2 - 0.6)	18.0% (9.5 - 24.9)	1.02 (1.02 - 1.02)
**Very preterm**	0.7 (0.5 - 1.0)	0.7 (0.4 - 0.9)	-0.1 (-0.2 - 0.0)	-8.7% (-6.8 - -10.3)	0.99 (0.99 - 1.00)
*Spontaneous*	0.4 (0.0 - 0.8)	0.5 (0.0 - 1.0)	0.1 (0.0 - 0.2)	22.7% (18.3 - 26.8)	1.01 (1.01 - 1.02)
*Provider-initiated*	0.4 (0.0 - 0.8)	0.2 (0.0 - 0.7)	-0.2 (-0.2 - -0.1)	-41.3% (-35.0 - -47.1)	0.96 (0.95 - 0.97)
**Extremely Preterm**	0.4 (0.2 - 0.6)	0.3 (0.1 - 0.6)	0.0 (-0.1 - 0.0)	-12.4% (-10.4 - -14.2)	0.99 (0.99 - 1.00)
*Spontaneous*	0.2 (0.0 - 0.7)	0.3 (0.0 - 0.7)	0.0 (-0.1 - 0.1)	5.4% (4.5 - 6.3)	1.01 (1.00 - 1.02)
*Provider-initiated*	0.1 (0.0 - 0.6)	0.1 (0.0 - 0.6)	-0.1 (-0.1 - 0.0)	-44.2% (-40.0 - -48.1)	0.96 (0.95 - 0.98)
**Term**	90.7 (90.4 - 90.9)	91.2 (91.2 - 91.2)	0.5 (0.2 - 0.9)	0.6% (-0.8 - 1.5)	1.01 (1.01 - 1.01)
**Post-term**	2.8 (2.6 - 3.0)	1.6 (1.6 - 1.6)	-1.2 (-1.4 - -1.0)	-42.3% (-23.6 - -56.5)	0.95 (0.95 - 0.95)

**Table:** (b) Live multiple births

	Percentage of all live multiple births in stated category, 2005	Percentage of all live multiple births in stated category, 2019	Absolute difference 2019 v 2005 (95% CI)	Relative (%) difference 2019 v 2005 (95% CI) *	Unadjusted odds ratio (95% CI)
**All Preterm**	57.6 (54.0 - 61.3)	63.5 (59.9 - 67.3)	6.0 (0.8 - 11.1)	10.3 (-6.4 - 16.2)	1.05 (1.04 - 1.06)
*Spontaneous*	26.6 (22.8 - 30.5)	27.3 (23.3 - 31.5)	0.8 (-3.9 - 5.5)	2.9 (-2.4 - 5.2)	1.01 (1.00 - 1.02)
*Provider-initiated*	31.0 (27.2 - 34.9)	36.1 (32.1 - 40.2)	5.1 (0.1 - 10.1)	16.5 (-8.2 - 26.5)	1.04 (1.03 - 1.05)
**Moderate to late preterm**	45.2 (41.5 - 49.1)	54.3 (50.6 - 58.3)	9.1 (3.9 - 14.4)	20.2 (-11.3 - 31.1)	1.03 (1.02 - 1.04)
*Spontaneous*	19.0 (15.4 - 22.8)	21.2 (17.3 - 25.2)	2.1 (-2.1 - 6.4)	11.3 ( -3.8 - 18.9)	1.01 (1.00 - 1.02)
*Provider-initiated*	26.2 (22.5 - 29.9)	33.1 (29.2 - 37.2)	6.9 (2.1 - 11.7)	26.4 (-10.9 - 43.0)	1.05 (1.04 - 1.06)
**Very preterm**	8.7 (5.1 - 12.6)	7.2 (3.4 - 11.2)	-1.5 (-4.4 - 1.4)	-17.7 ( -3.5 - -26.6)	1.00 (0.98 - 1.01)
*Spontaneous*	4.8 (1.2 - 8.6)	4.3 (0.4 - 8.4)	-0.5 (-2.8 - 1.8)	-10.4 ( -4.3 - -14.8)	1.01 (0.99 - 1.03)
*Provider-initiated*	3.9 (0.3 - 7.6)	2.9 (0.0 - 6.9)	-1.0 (-3.0 - 1.0)	-26.3 (-12.2 - -36.8)	0.98 (0.96 - 1.01)
**Extremely Preterm**	3.6 (0.0 - 7.5)	2.0 (0.0 - 6.0)	-1.6 (-3.5 - 0.2)	-44.8 (-22.2 - -61.4)	0.99 (0.97 - 1.02)
*Spontaneous*	2.7 (0.0 - 6.5)	1.9 (0.0 - 5.9)	-0.9 (-2.5 - 0.8)	-31.6 (-17.6 - -42.4)	1.00 (0.97 - 1.03)
*Provider-initiated*	0.9 (0.0 - 4.7)	0.1 (0.0 - 4.2)	-0.8 (-1.6 - 0.1)	-84.2 (-66.1 - -99.4)	0.98 (0.92 - 1.03)
**Term**	42.4 (38.9 - 46.2)	36.5 (32.9 - 40.3)	-6.0 (-11.1 - -0.8)	-14.0 (5.1 - -20.8)	0.96 (0.95 - 0.96)

Table 3a shows the percentage of all live singleton births in Scotland in 2005 and 2019 that were in different gestational age (and, for preterm births, different onset of labour) categories. The absolute and relative difference in the percentage of births that were preterm, term, and post-term in 2019 compared to 2005 is shown. Odds ratios indicating the estimated average change per year in the unadjusted odds of a live singleton birth being preterm, term and post-term are also shown. Table 3b shows similar information for live multiple births. Results not shown for post-term multiple births due to small numbers CI Confidence Interval *CI for relative difference calculated using the method in Kohavi *et al.*, 2009 ^31^ Gestational age categories are: preterm <37 weeks (moderate to late preterm 32–36 weeks; very preterm 28–31 weeks; extremely preterm <28 weeks); term 37–41 weeks; post term ≥42 weeks

***Category of preterm birth.*** Over the total study period, 42,424 (5.3%) of all singleton births were moderate to late preterm; 5,618 (0.7%) were very preterm; and 2,904 (0.4%) were extremely preterm ( Table 4).

**Table 4.** Number (%) of preterm births in different gestation and onset of labour categories, Scotland 2005–2019. (a) Live singleton births

Singleton births		Total 2005–2019	Year															Absolute difference 2019 v 2005	Relative (%) difference 2019 v 2005
			2005	2006	2007	2008	2009	2010	2011	2012	2013	2014	2015	2016	2017	2018	2019		
**All preterm**	Number	50946	3361	3311	3409	3669	3476	3268	3322	3296	3235	3305	3546	3463	3481	3396	3408	47	1.4%
	%	6.4	6.5	6.3	6.1	6.4	6.2	5.8	5.9	5.9	6.0	6.1	6.7	6.6	6.9	6.9	7.2	0.7	10.3%
*Spontaneous*	*Number*	30658	2000	2022	2088	2205	2106	2020	2036	2017	1927	1961	2050	2040	2062	2053	2071	71	3.6%
	*%*	3.8	3.9	3.8	3.8	3.9	3.7	3.6	3.6	3.6	3.6	3.6	3.9	3.9	4.1	4.2	4.4	0.5	12.7%
*Provider-initiated*	*Number*	20256	1360	1289	1320	1463	1370	1247	1285	1278	1307	1344	1493	1421	1417	1335	1327	-33	-2.4%
	*%*	2.5	2.6	2.4	2.4	2.6	2.4	2.2	2.3	2.3	2.4	2.5	2.8	2.7	2.8	2.7	2.8	0.2	6.2%
**Moderate to** ** late preterm**	Number	42424	2768	2730	2776	3042	2874	2674	2703	2730	2704	2719	3026	2947	2943	2871	2917	149	5.4%
	%	5.3	5.4	5.2	5.0	5.3	5.1	4.7	4.8	4.9	5.0	5.0	5.7	5.6	5.8	5.8	6.1	0.8	14.6%
*Spontaneous*	*Number*	25491	1675	1711	1730	1853	1755	1677	1673	1688	1601	1602	1724	1706	1686	1685	1725	50	3.0%
	*%*	3.2	3.2	3.2	3.1	3.2	3.1	3.0	3.0	3.0	3.0	2.9	3.3	3.3	3.3	3.4	3.6	0.4	12.1%
*Provider-initiated*	*Number*	16916	1093	1019	1045	1189	1119	996	1030	1042	1103	1117	1300	1241	1255	1182	1185	92	8.4%
	*%*	2.1	2.1	1.9	1.9	2.1	2.0	1.8	1.8	1.9	2.1	2.1	2.5	2.4	2.5	2.4	2.5	0.4	18.0%
**Very preterm**	Number	5618	387	384	406	425	395	376	431	387	374	382	328	339	328	351	325	-62	-16.0%
	%	0.7	0.7	0.7	0.7	0.7	0.7	0.7	0.8	0.7	0.7	0.7	0.6	0.6	0.6	0.7	0.7	-0.1	-8.7%
*Spontaneous*	*Number*	3267	196	196	211	235	216	206	250	218	227	215	191	216	224	245	221	25	12.8%
	*%*	0.4	0.4	0.4	0.4	0.4	0.4	0.4	0.4	0.4	0.4	0.4	0.4	0.4	0.4	0.5	0.5	0.1	22.7%
*Provider-initiated*	*Number*	2347	191	188	195	190	179	170	181	169	147	167	136	123	104	104	103	-88	-46.1%
	*%*	0.3	0.4	0.4	0.4	0.3	0.3	0.3	0.3	0.3	0.3	0.3	0.3	0.2	0.2	0.2	0.2	-0.2	-41.3%
**Extremely ** **preterm**	Number	2904	206	197	227	202	207	218	188	179	157	204	192	177	210	174	166	-40	-19.4%
	%	0.4	0.4	0.4	0.4	0.4	0.4	0.4	0.3	0.3	0.3	0.4	0.4	0.3	0.4	0.4	0.3	0.0	-12.4%
*Spontaneous*	*Number*	1900	129	115	147	117	135	137	113	111	99	144	135	118	152	123	125	-4	-3.1%
	*%*	0.2	0.2	0.2	0.3	0.2	0.2	0.2	0.2	0.2	0.2	0.3	0.3	0.2	0.3	0.2	0.3	0.0	5.4%
*Provider-initiated*	*Number*	993	76	82	80	84	72	81	74	67	57	60	57	57	58	49	39	-37	-48.7%
	*%*	0.1	0.1	0.2	0.1	0.1	0.1	0.1	0.1	0.1	0.1	0.1	0.1	0.1	0.1	0.1	0.1	-0.1	-44.2%

**Table:** (b) Live multiple births

Multiple births		Total 2005–2019	Year															Absolute difference 2019 v 2005	Relative (%) difference 2019 v 2005
			2005	2006	2007	2008	2009	2010	2011	2012	2013	2014	2015	2016	2017	2018	2019		
**All preterm**	Number	7158	442	431	458	506	453	490	465	468	472	502	509	530	507	483	442	0	0.0%
	%	58.7	57.6	53.5	52.8	54.5	50.4	54.1	56.2	56.0	60.6	60.2	64.7	67.7	68.3	65.2	63.5	6.0	10.3%
*Spontaneous*	*Number*	3073	204	195	208	225	215	224	201	185	192	195	197	237	211	194	190	-14	-6.9%
	*%*	25.2	26.6	24.2	24.0	24.2	23.9	24.8	24.3	22.1	24.6	23.4	25.0	30.3	28.4	26.3	27.3	0.8	2.9%
*Provider-initiated*	*Number*	4080	238	236	248	281	238	266	264	283	280	307	312	293	296	287	251	13	5.5%
	*%*	33.5	31.0	29.3	28.7	30.3	26.5	29.4	31.9	33.9	35.9	36.8	39.6	37.4	39.9	38.8	36.1	5.1	16.5%
**Moderate to** ** late preterm**	Number	5965	347	351	379	405	377	392	401	404	401	409	443	439	426	413	378	31	8.9%
	%	48.9	45.2	43.6	43.7	43.6	41.9	43.3	48.5	48.3	51.5	49.0	56.3	56.1	57.4	55.7	54.3	9.1	20.2%
*Spontaneous*	*Number*	2321	146	145	159	163	168	165	160	144	149	145	158	171	154	147	147	1	0.7%
	*%*	19.0	19.0	18.0	18.4	17.6	18.7	18.3	19.3	17.2	19.1	17.4	20.1	21.8	20.8	19.9	21.2	2.1	11.3%
*Provider-initiated*	*Number*	3639	201	206	218	242	209	227	241	260	252	264	285	268	272	264	230	29	14.4%
	*%*	29.8	26.2	25.6	25.2	26.1	23.2	25.1	29.1	31.1	32.3	31.7	36.2	34.2	36.7	35.7	33.1	6.9	26.4%
**Very preterm**	Number	884	67	55	64	72	60	75	49	46	51	66	49	69	64	47	50	-17	-25.4%
	%	7.2	8.7	6.8	7.4	7.8	6.7	8.3	5.9	5.5	6.5	7.9	6.2	8.8	8.6	6.3	7.2	-1.5	-17.7%
*Spontaneous*	*Number*	513	37	29	34	42	35	43	30	27	32	32	24	46	43	29	30	-7	-18.9%
	*%*	4.2	4.8	3.6	3.9	4.5	3.9	4.8	3.6	3.2	4.1	3.8	3.0	5.9	5.8	3.9	4.3	-0.5	-10.4%
*Provider-initiated*	*Number*	371	30	26	30	30	25	32	19	19	19	34	25	23	21	18	20	-10	-33.3%
	*%*	3.0	3.9	3.2	3.5	3.2	2.8	3.5	2.3	2.3	2.4	4.1	3.2	2.9	2.8	2.4	2.9	-1.0	-26.3%
**Extremely** ** preterm**	Number	309	28	25	15	29	16	23	15	18	20	27	17	22	17	23	14	-14	-50.0%
	%	2.5	3.6	3.1	1.7	3.1	1.8	2.5	1.8	2.2	2.6	3.2	2.2	2.8	2.3	3.1	2.0	-1.6	-44.8%
*Spontaneous*	*Number*	239	21	21	15	20	12	16	11	14	11	18	15	20	14	18	13	-8	-38.1%
	*%*	2.0	2.7	2.6	1.7	2.2	1.3	1.8	1.3	1.7	1.4	2.2	1.9	2.6	1.9	2.4	1.9	-0.9	-31.6%
*Provider-initiated*	*Number*	70	7	4	0	9	4	7	4	4	9	9	2	2	3	5	1	-6	-85.7%
	*%*	0.6	0.9	0.5	0.0	1.0	0.4	0.8	0.5	0.5	1.2	1.1	0.3	0.3	0.4	0.7	0.1	-0.8	-84.2%

For the categories including all onset types, %s are based on births with known gestation For the spontaneous and provider-initiated categories, %s are based on births with known gestation and onset of labour 32 live singleton preterm births had unknown onset of labour and are excluded from the spontaneous / provider-initiated figures 5 live multiple preterm births had unknown onset of labour and are excluded from the spontaneous / provider-initiated figures

**Figure 2.**
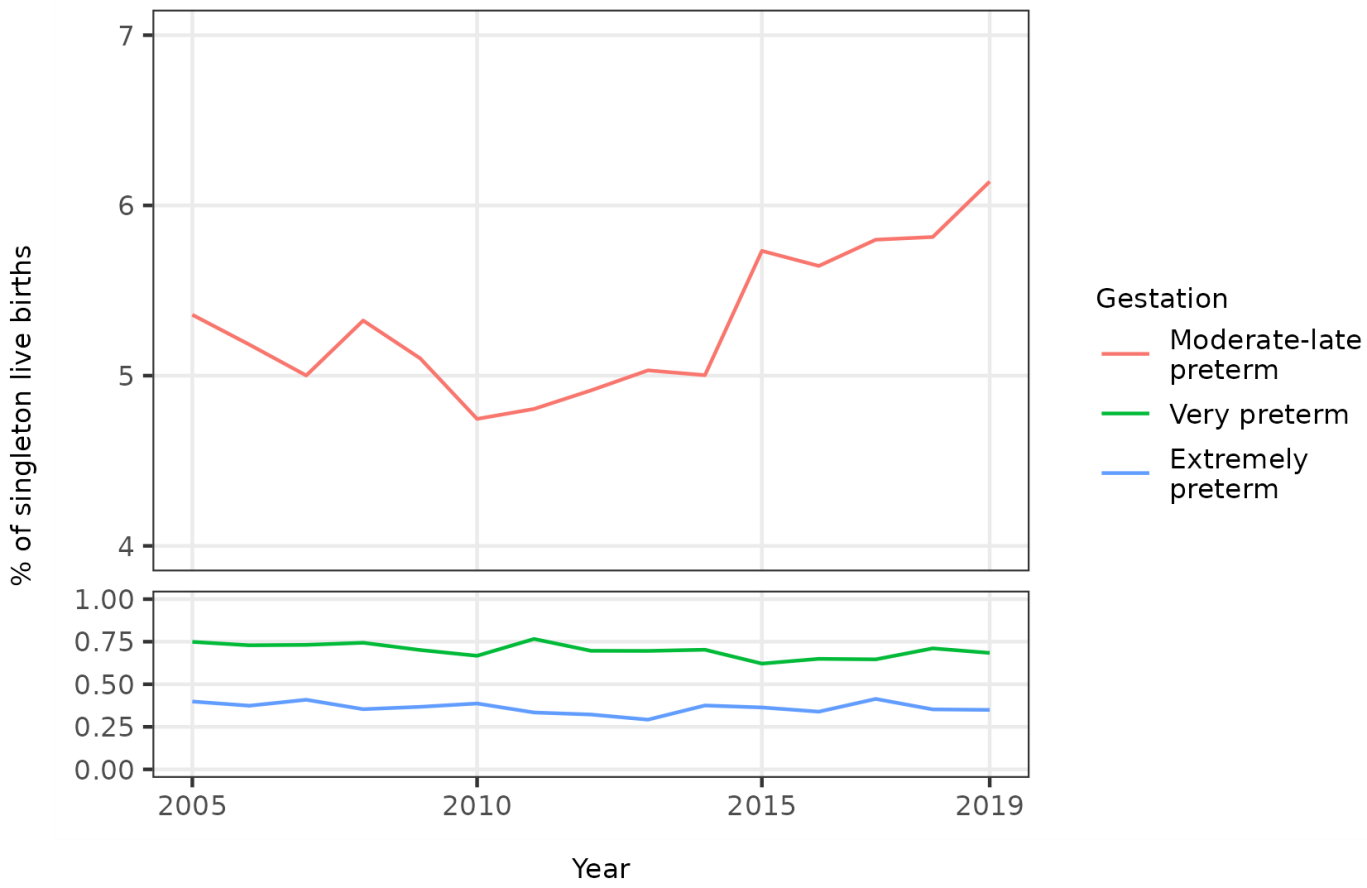
Trends in categories of preterm birth, singleton live births, Scotland. Figure 2 shows trends in the percentage of all live singleton births in Scotland between 2005 and 2019 that were moderate to late, very, and extremely preterm.

The percentage of all singleton births that were moderate to late preterm term decreased until 2010 before increasing until 2019 whilst the percentage that were very or extremely preterm was broadly static over the study period ( Figure 2, Table 4). The overall trends in the percentage of singleton births that were preterm were therefore driven by trends in the percentage that were moderate to late preterm. The percentage of all singleton births that were moderate to late preterm increased from 5.4% in 2005 to 6.1% in 2019. The unadjusted odds of a singleton birth being moderate to late preterm increased on average by 1% per year between 2005 and 2019 (OR 1.01, 95% CI 1.01 - 1.01) ( Table 3).

***Onset of labour.*** Over the total study period, 30,658 (60.2%) of all singleton preterm births were spontaneous and 20,256 (39.8%) were provider-initiated (with 32 having unknown onset of labour). Trends in the percentage of all singleton births that were spontaneous preterm and provider-initiated preterm showed similar trends over time, with an initial decrease to 2010 followed by an increase to 2019. The percentage of all singleton preterm births that were spontaneous was therefore broadly static at around 60% over the study period ( Table 4).

**Figure 3.**
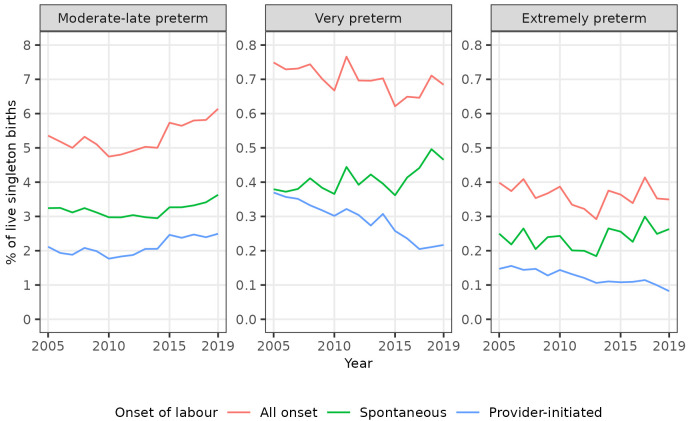
Trends in categories of preterm birth by onset of labour, singleton live births, Scotland. Figure 3 shows trends in the percentage of all live singleton births in Scotland between 2005 and 2019 that were moderate to late, very, and extremely preterm, by onset of labour (spontaneous or provider-initiated).

Trends in spontaneous and provider-initiated preterm births varied by category of preterm birth, however. The percentages of all singleton births that were spontaneous moderate to late preterm, spontaneous very preterm, and spontaneous extremely preterm all increased in the years leading up to 2019. Conversely, whilst the percentage of all singleton births that were provider-initiated moderate to late preterm also increased over this period, the percentages that were provider-initiated very preterm, and provider-initiated extremely preterm decreased ( Table 4, Figure 3). Consequently, whilst the percentage of singleton moderate to late preterm births that were spontaneous was broadly static at around 60% over the study period, the percentage of singleton very preterm births that were spontaneous increased from 50.6% (196 / 387) in 2005 to 68.2% (221 / 324) in 2019, and the percentage of singleton extremely preterm births that were spontaneous increased from 62.9% (129 / 205) in 2005 to 76.2% (125 / 164) in 2019 ( Table 4).

***Trends by maternal age and deprivation.*** There was a shift in the distribution of maternal age at birth over our study period, with the percentage of all singleton births to women aged <20 years decreasing from 7.9% (4,106 / 51,665) in 2005 to 3.0% (1,417 / 47,507) in 2019, and conversely the percentage to women aged ≥40 years increasing from 3.2% (1,635 / 51,665) in 2005 to 4.1% (1,925 / 47,507) in 2019 ( Table 5).

**Table 5.** Number (%) of births by maternal age category, Scotland 2005–2019. (a) Live singleton births

Singleton births Maternal age		Total 2005–2019	Year															Absolute difference 2019 v 2005	Relative (%) difference 2019 v 2005
			2005	2006	2007	2008	2009	2010	2011	2012	2013	2014	2015	2016	2017	2018	2019		
**<20**	Number	44677	4106	4039	4259	4211	3940	3733	3367	3067	2730	2407	2097	1915	1810	1579	1417	-2689	-65.5%
	%	5.6	7.9	7.7	7.7	7.4	7.0	6.6	6.0	5.5	5.1	4.4	4.0	3.7	3.6	3.2	3.0	-5.0	-62.5%
**20–24**	Number	139449	9765	9988	10701	10991	10884	10464	10329	10070	9502	9022	8466	8110	7562	7073	6522	-3243	-33.2%
	%	17.4	18.9	19.0	19.3	19.2	19.3	18.6	18.4	18.1	17.7	16.6	16.0	15.5	14.9	14.3	13.7	-5.2	-27.4%
**25–29**	Number	217511	12684	13260	14417	15537	15478	15562	15355	15427	14726	15198	14787	14447	14044	13603	12986	302	2.4%
	%	27.1	24.6	25.2	26.0	27.2	27.5	27.6	27.3	27.8	27.4	28.0	28.0	27.7	27.7	27.5	27.3	2.8	11.3%
**30–34**	Number	237275	15034	14868	14874	15091	15017	15610	16124	16366	16267	16875	16590	16707	16182	15928	15742	708	4.7%
	%	29.6	29.1	28.2	26.8	26.4	26.7	27.7	28.7	29.5	30.3	31.0	31.4	32.0	31.9	32.3	33.1	4.0	13.9%
**35–39**	Number	134082	8441	8845	9444	9329	9028	8994	8976	8573	8426	8802	8914	8983	9230	9182	8915	474	5.6%
	%	16.7	16.3	16.8	17.0	16.3	16.0	16.0	16.0	15.4	15.7	16.2	16.9	17.2	18.2	18.6	18.8	2.4	14.9%
**≥40**	Number	29222	1635	1676	1814	1982	1985	1981	2109	2059	2096	2046	1929	2048	1925	2012	1925	290	17.7%
	%	3.6	3.2	3.2	3.3	3.5	3.5	3.5	3.7	3.7	3.9	3.8	3.7	3.9	3.8	4.1	4.1	0.9	28.0%

**Table:** (b) Live multiple births

Multiple births Maternal age		Total 2005–2019	Year															Absolute difference 2019 v 2005	Relative (%) difference 2019 v 2005
			2005	2006	2007	2008	2009	2010	2011	2012	2013	2014	2015	2016	2017	2018	2019		
**<20**	Number	269	16	21	31	34	23	22	20	11	16	20	9	18	11	8	9	-7	-43.8%
	%	2.2	2.1	2.6	3.6	3.7	2.6	2.4	2.4	1.3	2.1	2.4	1.1	2.3	1.5	1.1	1.3	-0.8	-37.9%
**20–24**	Number	1255	72	90	101	111	106	90	87	89	92	79	67	64	72	71	64	-8	-11.1%
	%	10.3	9.4	11.2	11.6	11.9	11.8	9.9	10.5	10.7	11.8	9.5	8.5	8.2	9.7	9.6	9.2	-0.2	-1.9%
**25–29**	Number	2736	160	172	172	184	221	202	179	198	181	171	171	185	184	188	168	8	5.0%
	%	22.4	20.8	21.4	19.8	19.8	24.6	22.3	21.6	23.7	23.2	20.5	21.7	23.6	24.8	25.4	24.1	3.3	15.9%
**30–34**	Number	4133	277	275	275	316	268	297	272	298	263	280	293	274	246	246	253	-24	-8.7%
	%	33.9	36.1	34.2	31.7	34.0	29.8	32.8	32.9	35.7	33.8	33.6	37.2	35.0	33.2	33.2	36.4	0.3	0.8%
**35–39**	Number	2999	199	208	240	232	230	223	201	195	177	224	182	194	178	174	142	-57	-28.6%
	%	24.6	25.9	25.8	27.7	25.0	25.6	24.6	24.3	23.4	22.7	26.9	23.1	24.8	24.0	23.5	20.4	-5.5	-21.3%
**≥40**	Number	805	44	39	48	52	51	71	68	44	50	60	65	48	51	54	60	16	36.4%
	%	6.6	5.7	4.8	5.5	5.6	5.7	7.8	8.2	5.3	6.4	7.2	8.3	6.1	6.9	7.3	8.6	2.9	50.5%

% based on births with known gestation and maternal age 1 live singleton birth had known gestation but unknown maternal age 1 live multiple birth had known gestation but unknown maternal age

**Figure 4.**
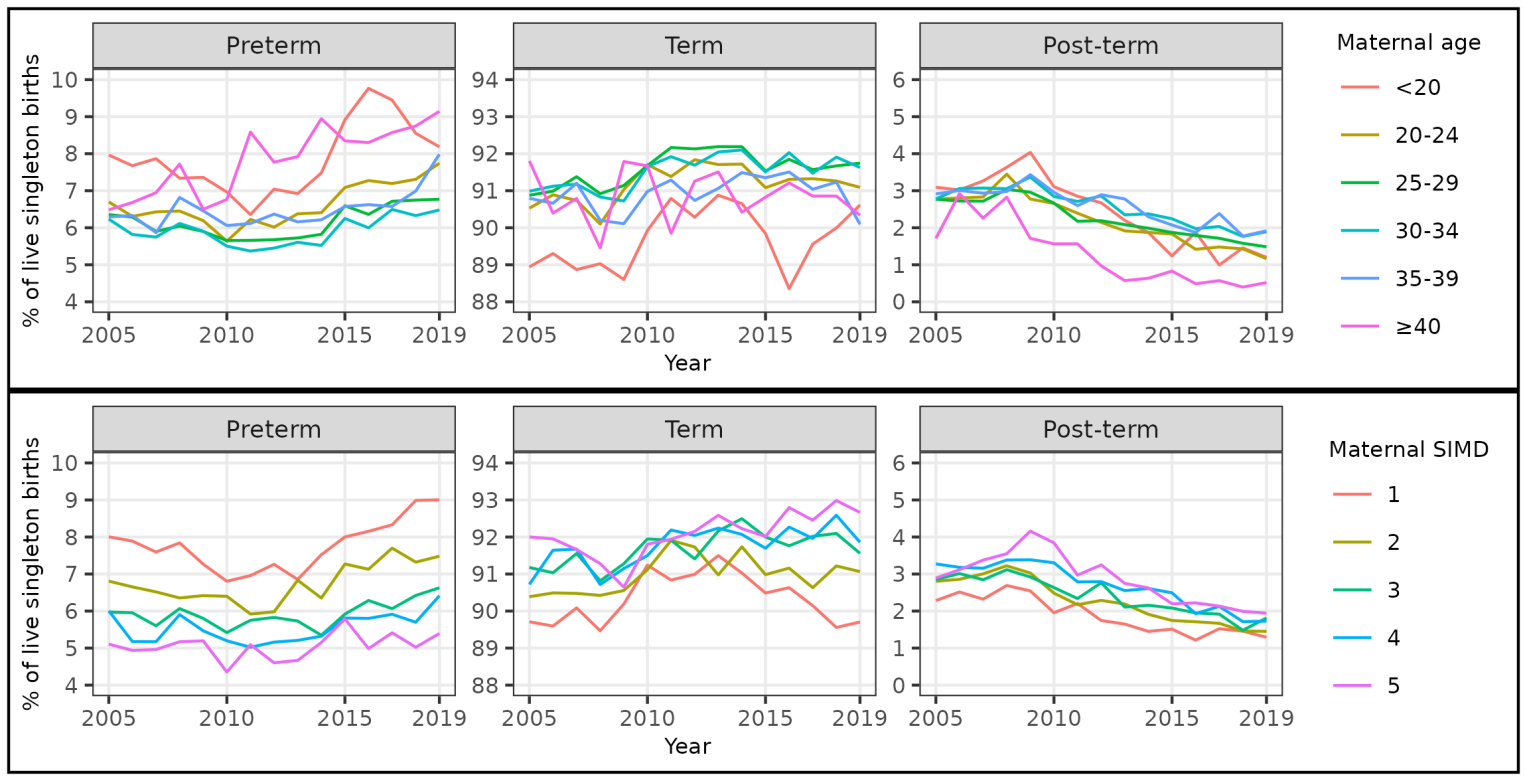
Trends in gestational age at birth by maternal age and deprivation, singleton live births, Scotland. **Figure 4a** shows trends in the percentage of all live singleton births in Scotland between 2005 and 2019 that were preterm, term, and post-term, by maternal age category. **Figure 4b** shows trends in the percentage of all live singleton births in Scotland between 2005 and 2019 that were preterm, term, and post-term, by maternal Scottish Index of Multiple Deprivation (SIMD) quintile.

Over the whole study period, a U-shaped relationship between maternal age and risk of preterm birth was evident. The percentage of singleton births that were preterm was highest amongst births to women aged <20 (7.7%, 3,426 / 44,677) and ≥40 (7.9%, 2,298 / 29,222), and lowest amongst births to women aged 30–34 (5.9%, 14,046 / 237,275) ( Table 6). The percentage of singleton births that were preterm increased in all maternal age categories between 2010 and 2019. The increase was most marked for women in the youngest and oldest age categories, hence the absolute difference in risk between women at the extremes of age compared to the intermediate age category increased over time ( Figure 4, Table 6, Table 7).

**Table 6.** Number (%) of births that were preterm, term, and post-term by maternal age, Scotland 2005–2019. (a) Live singleton births

Singleton births	Maternal age		Total 2005–2019	Year															Absolute difference 2019 v 2005	Relative (%) difference 2019 v 2005
				2005	2006	2007	2008	2009	2010	2011	2012	2013	2014	2015	2016	2017	2018	2019		
**Preterm**	**<20**	Number	3426	327	310	335	309	290	260	214	216	189	180	187	187	171	135	116	-211	-64.5%
		%	7.7	8.0	7.7	7.9	7.3	7.4	7.0	6.4	7.0	6.9	7.5	8.9	9.8	9.4	8.5	8.2	0.2	2.8%
	**20–24**	Number	9134	654	630	688	709	674	590	643	606	606	578	600	590	544	517	505	-149	-22.8%
		%	6.6	6.7	6.3	6.4	6.5	6.2	5.6	6.2	6.0	6.4	6.4	7.1	7.3	7.2	7.3	7.7	1.0	15.6%
	**25–29**	Number	13330	806	834	851	939	913	880	869	876	843	885	975	919	943	918	879	73	9.1%
		%	6.1	6.4	6.3	5.9	6.0	5.9	5.7	5.7	5.7	5.7	5.8	6.6	6.4	6.7	6.7	6.8	0.4	6.5%
	**30–34**	Number	14046	937	865	855	923	887	859	866	892	912	932	1037	1002	1051	1008	1020	83	8.9%
		%	5.9	6.2	5.8	5.7	6.1	5.9	5.5	5.4	5.5	5.6	5.5	6.3	6.0	6.5	6.3	6.5	0.2	4.0%
	**35–39**	Number	8712	531	560	554	636	583	545	549	546	519	547	586	595	607	642	712	181	34.1%
		%	6.5	6.3	6.3	5.9	6.8	6.5	6.1	6.1	6.4	6.2	6.2	6.6	6.6	6.6	7.0	8.0	1.7	27.0%
	**≥40**	Number	2298	106	112	126	153	129	134	181	160	166	183	161	170	165	176	176	70	66.0%
		%	7.9	6.5	6.7	6.9	7.7	6.5	6.8	8.6	7.8	7.9	8.9	8.3	8.3	8.6	8.7	9.1	2.7	41.0%
**Term**	**<20**	Number	40032	3652	3607	3785	3749	3491	3357	3057	2769	2481	2182	1884	1692	1621	1421	1284	-2368	-64.8%
		%	89.6	88.9	89.3	88.9	89.0	88.6	89.9	90.8	90.3	90.9	90.7	89.8	88.4	89.6	90.0	90.6	1.7	1.9%
	**20–24**	Number	127129	8840	9078	9710	9903	9908	9596	9439	9248	8714	8275	7711	7405	6906	6455	5941	-2899	-32.8%
		%	91.2	90.5	90.9	90.7	90.1	91.0	91.7	91.4	91.8	91.7	91.7	91.1	91.3	91.3	91.3	91.1	0.6	0.6%
	**25–29**	Number	199266	11527	12065	13174	14126	14107	14267	14152	14213	13576	14011	13535	13269	12860	12470	11914	387	3.4%
		%	91.6	90.9	91.0	91.4	90.9	91.1	91.7	92.2	92.1	92.2	92.2	91.5	91.8	91.6	91.7	91.7	0.9	1.0%
	**30–34**	Number	217190	13679	13548	13562	13707	13624	14307	14821	15006	14973	15542	15181	15375	14802	14639	14424	745	5.4%
		%	91.5	91.0	91.1	91.2	90.8	90.7	91.7	91.9	91.7	92.0	92.1	91.5	92.0	91.5	91.9	91.6	0.6	0.7%
	**35–39**	Number	121903	7664	8019	8613	8415	8135	8183	8194	7779	7673	8053	8143	8220	8403	8377	8032	368	4.8%
		%	90.9	90.8	90.7	91.2	90.2	90.1	91.0	91.3	90.7	91.1	91.5	91.4	91.5	91.0	91.2	90.1	-0.7	-0.8%
	**≥40**	Number	26552	1501	1515	1647	1773	1822	1816	1895	1879	1918	1850	1752	1868	1749	1828	1739	238	15.9%
		%	90.9	91.8	90.4	90.8	89.5	91.8	91.7	89.9	91.3	91.5	90.4	90.8	91.2	90.9	90.9	90.3	-1.5	-1.6%
**Post-term**	**<20**	Number	1219	127	122	139	153	159	116	96	82	60	45	26	36	18	23	17	-110	-86.6%
		%	2.7	3.1	3.0	3.3	3.6	4.0	3.1	2.9	2.7	2.2	1.9	1.2	1.9	1.0	1.5	1.2	-1.9	-61.2%
	**20–24**	Number	3186	271	280	303	379	302	278	247	216	182	169	155	115	112	101	76	-195	-72.0%
		%	2.3	2.8	2.8	2.8	3.4	2.8	2.7	2.4	2.1	1.9	1.9	1.8	1.4	1.5	1.4	1.2	-1.6	-58.0%
	**25–29**	Number	4915	351	361	392	472	458	415	334	338	307	302	277	259	241	215	193	-158	-45.0%
		%	2.3	2.8	2.7	2.7	3.0	3.0	2.7	2.2	2.2	2.1	2.0	1.9	1.8	1.7	1.6	1.5	-1.3	-46.3%
	**30–34**	Number	6039	418	455	457	461	506	444	437	468	382	401	372	330	329	281	298	-120	-28.7%
		%	2.5	2.8	3.1	3.1	3.1	3.4	2.8	2.7	2.9	2.3	2.4	2.2	2.0	2.0	1.8	1.9	-0.9	-31.9%
	**35–39**	Number	3467	246	266	277	278	310	266	233	248	234	202	185	168	220	163	171	-75	-30.5%
		%	2.6	2.9	3.0	2.9	3.0	3.4	3.0	2.6	2.9	2.8	2.3	2.1	1.9	2.4	1.8	1.9	-1.0	-34.2%
	**≥40**	Number	372	28	49	41	56	34	31	33	20	12	13	16	10	11	8	10	-18	-64.3%
		%	1.3	1.7	2.9	2.3	2.8	1.7	1.6	1.6	1.0	0.6	0.6	0.8	0.5	0.6	0.4	0.5	-1.2	-69.7%

**Table:** (b) Live multiple births

Multiple births	Maternal age		Total 2005–2019	Year															Absolute difference 2019 v 2005	Relative (%) difference 2019 v 2005
				2005	2006	2007	2008	2009	2010	2011	2012	2013	2014	2015	2016	2017	2018	2019		
**Preterm**	**<20**	Number	186	11	14	15	24	15	17	9	7	12	14	6	18	10	7	7	-4	-36.4%
		%	69.1	68.8	66.7	48.4	70.6	65.2	77.3	45.0	63.6	75.0	70.0	66.7	100.0	90.9	87.5	77.8	9.0	13.1%
	**20–24**	Number	786	44	50	58	70	60	54	46	51	54	54	42	50	54	51	48	4	9.1%
		%	62.6	61.1	55.6	57.4	63.1	56.6	60.0	52.9	57.3	58.7	67.5	61.8	78.1	75.0	71.8	75.0	13.9	22.7%
	**25–29**	Number	1672	99	102	97	99	109	115	104	112	118	115	110	124	136	118	114	15	15.2%
		%	61.1	61.9	59.3	56.4	53.8	49.3	56.9	58.1	56.6	65.2	66.5	64.0	66.7	73.9	62.8	67.9	6.0	9.7%
	**30–34**	Number	2362	162	144	142	159	129	152	160	159	157	159	192	175	156	157	159	-3	-1.9%
		%	57.1	58.3	52.4	51.6	50.3	48.0	51.2	58.8	53.4	59.7	56.8	65.3	63.2	62.7	63.8	62.8	4.4	7.5%
	**35–39**	Number	1690	101	104	127	123	109	112	110	107	104	128	122	135	116	114	78	-23	-22.8%
		%	56.4	50.8	50.0	52.9	53.0	47.4	50.2	54.7	54.9	58.8	57.1	67.0	68.5	65.2	65.5	54.9	4.2	8.2%
	**≥40**	Number	462	25	17	19	31	31	40	36	32	27	32	37	28	35	36	36	11	44.0%
		%	57.4	56.8	43.6	39.6	59.6	60.8	56.3	52.9	72.7	54.0	53.3	56.9	58.3	68.6	66.7	60.0	3.2	5.6%
**Term**	**<20**	Number	83	5	7	16	10	8	5	11	4	4	6	3	0	1	1	2	-3	-60.0%
		%	30.9	31.3	33.3	51.6	29.4	34.8	22.7	55.0	36.4	25.0	30.0	33.3	0.0	9.1	12.5	22.2	-9.0	-28.9%
	**20–24**	Number	469	28	40	43	41	46	36	41	38	38	25	25	14	18	20	16	-12	-42.9%
		%	37.4	38.9	44.4	42.6	36.9	43.4	40.0	47.1	42.7	41.3	31.3	36.8	21.9	25.0	28.2	25.0	-13.9	-35.7%
	**25–29**	Number	1064	61	70	75	85	112	87	75	86	63	56	61	61	48	70	54	-7	-11.5%
		%	38.9	38.1	40.7	43.6	46.2	50.7	43.1	41.9	43.4	34.8	32.4	35.5	32.8	26.1	37.2	32.1	-6.0	-15.7%
	**30–34**	Number	1770	115	130	133	157	139	145	112	139	106	121	101	99	90	89	94	-21	-18.3%
		%	42.8	41.4	47.3	48.4	49.7	51.7	48.8	41.2	46.6	40.3	43.2	34.4	35.7	36.1	36.2	37.2	-4.4	-10.5%
	**35–39**	Number	1308	98	104	112	109	121	111	91	88	73	96	60	59	62	60	64	-34	-34.7%
		%	43.6	49.2	50.0	46.7	47.0	52.6	49.8	45.3	45.1	41.2	42.9	33.0	29.9	34.8	34.5	45.1	-4.2	-8.5%
	**≥40**	Number	343	19	22	29	21	20	31	32	12	23	28	28	20	16	18	24	5	26.3%
		%	42.6	43.2	56.4	60.4	40.4	39.2	43.7	47.1	27.3	46.0	46.7	43.1	41.7	31.4	33.3	40.0	-3.2	-7.4%

% based on births with known gestation and maternal age 1 live singleton birth had known gestation but unknown maternal age 1 live multiple birth had known gestation but unknown maternal age Results not shown for post-term multiple births due to small numbers

**Table 7.** Preterm births to women in the oldest and intermediate age categories, Scotland 2005–2019. (a) Percentage of live singleton births to women in the oldest (≥40) and intermediate (30–34) age categories that were preterm, and absolute and relative difference between oldest and intermediate age categories, Scotland 2005–2019

Singleton preterm births Maternal age	Year														
	2005	2006	2007	2008	2009	2010	2011	2012	2013	2014	2015	2016	2017	2018	2019
**30–34**	6.2	5.8	5.7	6.1	5.9	5.5	5.4	5.5	5.6	5.5	6.3	6.0	6.5	6.3	6.5
**≥40**	6.5	6.7	6.9	7.7	6.5	6.8	8.6	7.8	7.9	8.9	8.3	8.3	8.6	8.7	9.1
**Absolute difference** **≥40–30–34**	0.3	0.9	1.2	1.6	0.6	1.3	3.2	2.3	2.3	3.4	2.1	2.3	2.1	2.4	2.7
**Ratio** **≥40:30–34**	1.0	1.1	1.2	1.3	1.1	1.2	1.6	1.4	1.4	1.6	1.3	1.4	1.3	1.4	1.4

**Table:** (b) Percentage of live multiple births to women in the oldest (≥40) and intermediate (30–34) age categories that were preterm, and absolute and relative difference between oldest and intermediate age categories, Scotland 2005–2019

Multiple births Maternal age	Year														
	2005	2006	2007	2008	2009	2010	2011	2012	2013	2014	2015	2016	2017	2018	2019
**30–34**	58.3	52.4	51.6	50.3	48.0	51.2	58.8	53.4	59.7	56.8	65.3	63.2	62.7	63.8	62.8
**≥40**	56.8	43.6	39.6	59.6	60.8	56.3	52.9	72.7	54.0	53.3	56.9	58.3	68.6	66.7	60.0
**Absolute difference** **≥40–30–34**	-1.5	-8.8	-12.1	9.3	12.8	5.2	-5.9	19.4	-5.7	-3.5	-8.4	-4.8	6.0	2.8	-2.8
**Ratio** **≥40:30–34**	1.0	0.8	0.8	1.2	1.3	1.1	0.9	1.4	0.9	0.9	0.9	0.9	1.1	1.0	1.0

Over the whole study period, the percentages of singleton births that were post-term were broadly similar among women aged <20 to 35–39 at around 2.5%, whilst the percentage was lower in women aged ≥40 at 1.3% (372 / 29,222) ( Table 6). The percentage of singleton births that were post-term decreased in all maternal age categories between 2009 and 2019 ( Figure 4, Table 6).

Over our study period, 25.6% (204,992 / 799,457) of singleton births were to women in the most deprived quintile and 16.5% (131,830 / 799,457) were to women in the least deprived quintile. The distribution of singleton births by maternal deprivation category was broadly static over time ( Table 8).

**Table 8.** Number (%) of births by maternal deprivation category, Scotland 2005–2019. (a) Live singleton births

Singleton births Maternal SIMD quintile		Total 2005–2019	Year															Absolute difference 2019 v 2005	Relative (%) difference 2019 v 2005
			2005	2006	2007	2008	2009	2010	2011	2012	2013	2014	2015	2016	2017	2018	2019		
**1 = most** **deprived**	Number	204992	12955	13119	14352	14679	14465	14725	14919	14665	13950	13920	13298	13086	13002	12095	11762	-1193	-9.2%
	%	25.6	25.1	25.0	25.9	25.8	25.8	26.2	26.6	26.5	26.1	25.7	25.3	25.1	25.7	24.5	24.8	-0.3	-1.3%
**2**	Number	168510	10341	10778	11415	11977	11827	11876	11926	11889	11415	11399	11347	10927	10663	10545	10185	-156	-1.5%
	%	21.1	20.1	20.5	20.6	21.0	21.1	21.2	21.3	21.5	21.4	21.0	21.6	21.0	21.1	21.4	21.5	1.4	7.0%
**3**	Number	148807	9611	9893	10123	10714	10824	10741	10694	10568	10156	9967	9512	9482	9183	8845	8494	-1117	-11.6%
	%	18.6	18.7	18.9	18.3	18.8	19.3	19.1	19.1	19.1	19.0	18.4	18.1	18.2	18.1	17.9	17.9	-0.7	-4.0%
**4**	Number	145318	9440	9621	10170	10298	10044	9987	9794	9551	9485	9811	9429	9635	9404	9510	9139	-301	-3.2%
	%	18.2	18.3	18.3	18.4	18.1	17.9	17.8	17.5	17.3	17.7	18.1	17.9	18.5	18.6	19.3	19.3	1.0	5.2%
**5 = least** **deprived**	Number	131830	9164	9055	9272	9301	8873	8797	8683	8625	8442	9106	9054	8964	8388	8282	7824	-1340	-14.6%
	%	16.5	17.8	17.3	16.8	16.3	15.8	15.7	15.5	15.6	15.8	16.8	17.2	17.2	16.6	16.8	16.5	-1.3	-7.2%

**Table:** (b) Live multiple births

Multiple births Maternal SIMD quintile		Total 2005–2019	Year															Absolute difference 2019 v 2005	Relative (%) difference 2019 v 2005
			2005	2006	2007	2008	2009	2010	2011	2012	2013	2014	2015	2016	2017	2018	2019		
**1 = most** **deprived**	Number	2640	136	159	177	208	209	199	167	182	175	184	181	178	160	171	154	18	13.2%
	%	21.7	17.8	19.8	20.6	22.5	23.5	22.1	20.3	21.9	22.6	22.2	23.1	22.8	21.6	23.2	22.3	4.6	25.7%
**2**	Number	2422	148	168	174	177	190	167	162	163	168	158	134	147	157	163	146	-2	-1.4%
	%	20.0	19.3	20.9	20.2	19.1	21.3	18.5	19.7	19.6	21.7	19.0	17.1	18.8	21.2	22.1	21.2	1.8	9.5%
**3**	Number	2311	144	151	172	162	144	171	161	169	139	178	160	145	143	144	128	-16	-11.1%
	%	19.0	18.8	18.8	20.0	17.5	16.2	19.0	19.6	20.3	17.9	21.4	20.4	18.6	19.3	19.5	18.6	-0.2	-1.3%
**4**	Number	2368	164	171	170	180	175	191	152	150	150	159	152	141	142	131	140	-24	-14.6%
	%	19.5	21.4	21.3	19.7	19.4	19.6	21.2	18.5	18.0	19.4	19.2	19.4	18.1	19.2	17.8	20.3	-1.1	-5.2%
**5 = least** **deprived**	Number	2399	174	153	168	199	173	174	181	168	143	151	157	169	139	128	122	-52	-29.9%
	%	19.8	22.7	19.1	19.5	21.5	19.4	19.3	22.0	20.2	18.5	18.2	20.0	21.7	18.8	17.4	17.7	-5.0	-22.2%

% based on births with known gestation and maternal SIMD quintile 2760 live singleton births had known gestation but unknown maternal SIMD quintile 58 live multiple births had known gestation but unknown maternal SIMD quintile

Over the whole study period, there was a clear deprivation gradient in risk of preterm birth. The percentage of singleton births that were preterm was highest amongst births to women in the most deprived quintile (SIMD quintile 1: 7.7%, 15,832 / 204,992) and lowest amongst births to women in the least deprived quintile (SIMD quintile 5: 5.1%, 6,665 / 131,830) ( Table 9). The percentage of singleton births that were preterm increased in all maternal deprivation categories between 2010 and 2019. The increase was most marked for women in the most deprived quintile, hence the absolute difference in risk between women in the most compared to the least deprived categories increased over time ( Figure 4, Table 9, Table 10).

**Table 9.** Number (%) of births that were preterm, term, and post-term by maternal deprivation, Scotland 2005–2019. (a) Live singleton births

Singleton births	Maternal SIMD quintile		Total 2005–2019	Year															Absolute difference 2019 v 2005	Relative (%) difference 2019 v 2005
				2005	2006	2007	2008	2009	2010	2011	2012	2013	2014	2015	2016	2017	2018	2019		
**Preterm**	**1 = most** **deprived**	Number	15832	1037	1035	1090	1151	1051	1002	1038	1065	956	1047	1064	1067	1083	1087	1059	22	2.1%
		%	7.7	8.0	7.9	7.6	7.8	7.3	6.8	7.0	7.3	6.9	7.5	8.0	8.2	8.3	9.0	9.0	1.0	12.5%
	**2**	Number	11325	704	717	744	761	759	760	706	711	780	724	825	779	821	772	762	58	8.2%
		%	6.7	6.8	6.7	6.5	6.4	6.4	6.4	5.9	6.0	6.8	6.4	7.3	7.1	7.7	7.3	7.5	0.7	9.9%
	**3**	Number	8783	574	589	567	650	628	582	615	616	582	533	563	596	557	568	563	-11	-1.9%
		%	5.9	6.0	6.0	5.6	6.1	5.8	5.4	5.8	5.8	5.7	5.3	5.9	6.3	6.1	6.4	6.6	0.7	11.0%
	**4**	Number	8059	567	498	526	608	549	519	492	493	494	522	548	559	556	542	586	19	3.4%
		%	5.5	6.0	5.2	5.2	5.9	5.5	5.2	5.0	5.2	5.2	5.3	5.8	5.8	5.9	5.7	6.4	0.4	6.8%
	**5 = least** **deprived**	Number	6665	468	447	460	481	461	383	442	397	394	469	524	447	454	416	422	-46	-9.8%
		%	5.1	5.1	4.9	5.0	5.2	5.2	4.4	5.1	4.6	4.7	5.2	5.8	5.0	5.4	5.0	5.4	0.3	5.6%
**Term**	**1 = most** **deprived**	Number	185247	11622	11754	12929	13133	13046	13435	13552	13344	12764	12671	12033	11860	11721	10832	10551	-1071	-9.2%
		%	90.4	89.7	89.6	90.1	89.5	90.2	91.2	90.8	91.0	91.5	91.0	90.5	90.6	90.1	89.6	89.7	0.0	0.0%
	**2**	Number	153341	9347	9753	10328	10830	10710	10821	10961	10906	10385	10457	10324	9961	9664	9619	9275	-72	-0.8%
		%	91.0	90.4	90.5	90.5	90.4	90.6	91.1	91.9	91.7	91.0	91.7	91.0	91.2	90.6	91.2	91.1	0.7	0.7%
	**3**	Number	136416	8763	9006	9268	9730	9880	9876	9829	9660	9360	9219	8751	8701	8450	8146	7777	-986	-11.3%
		%	91.7	91.2	91.0	91.6	90.8	91.3	91.9	91.9	91.4	92.2	92.5	92.0	91.8	92.0	92.1	91.6	0.4	0.4%
	**4**	Number	133325	8564	8817	9323	9342	9155	9138	9029	8791	8749	9033	8646	8890	8648	8805	8395	-169	-2.0%
		%	91.7	90.7	91.6	91.7	90.7	91.1	91.5	92.2	92.0	92.2	92.1	91.7	92.3	92.0	92.6	91.9	1.1	1.3%
	**5 = least** **deprived**	Number	121365	8431	8326	8499	8490	8043	8076	7983	7948	7816	8398	8331	8318	7755	7701	7250	-1181	-14.0%
		%	92.1	92.0	91.9	91.7	91.3	90.6	91.8	91.9	92.2	92.6	92.2	92.0	92.8	92.5	93.0	92.7	0.7	0.7%
**Post-** **term**	**1 = most** **deprived**	Number	3913	296	330	333	395	368	288	329	256	230	202	201	159	198	176	152	-144	-48.6%
		%	1.9	2.3	2.5	2.3	2.7	2.5	2.0	2.2	1.7	1.6	1.5	1.5	1.2	1.5	1.5	1.3	-1.0	-43.4%
	**2**	Number	3844	290	308	343	386	358	295	259	272	250	218	198	187	178	154	148	-142	-49.0%
		%	2.3	2.8	2.9	3.0	3.2	3.0	2.5	2.2	2.3	2.2	1.9	1.7	1.7	1.7	1.5	1.5	-1.4	-48.2%
	**3**	Number	3608	274	298	288	334	316	283	250	292	214	215	198	185	176	131	154	-120	-43.8%
		%	2.4	2.9	3.0	2.8	3.1	2.9	2.6	2.3	2.8	2.1	2.2	2.1	2.0	1.9	1.5	1.8	-1.0	-36.4%
	**4**	Number	3934	309	306	321	348	340	330	273	267	242	256	235	186	200	163	158	-151	-48.9%
		%	2.7	3.3	3.2	3.2	3.4	3.4	3.3	2.8	2.8	2.6	2.6	2.5	1.9	2.1	1.7	1.7	-1.5	-47.2%
	**5 = least** **deprived**	Number	3800	265	282	313	330	369	338	258	280	232	239	199	199	179	165	152	-113	-42.6%
		%	2.9	2.9	3.1	3.4	3.5	4.2	3.8	3.0	3.2	2.7	2.6	2.2	2.2	2.1	2.0	1.9	-0.9	-32.8%

**Table:** b) Live multiple births

Multiple births	Maternal SIMD quintile		Total 2005–2019	Year															Absolute difference 2019 v 2005	Relative (%) difference 2019 v 2005
				2005	2006	2007	2008	2009	2010	2011	2012	2013	2014	2015	2016	2017	2018	2019		
**Preterm**	**1 = most** **deprived**	Number	1631	80	91	107	128	107	114	103	94	104	121	121	126	114	115	106	26	32.5%
		%	61.8	58.8	57.2	60.5	61.5	51.2	57.3	61.7	51.6	59.4	65.8	66.9	70.8	71.3	67.3	68.8	10.0	17.0%
	**2**	Number	1436	92	90	93	104	94	89	86	89	97	95	85	108	110	108	96	4	4.3%
		%	59.3	62.2	53.6	53.4	58.8	49.5	53.3	53.1	54.6	57.7	60.1	63.4	73.5	70.1	66.3	65.8	3.6	5.8%
	**3**	Number	1335	76	73	83	87	76	83	92	108	88	100	103	93	91	102	80	4	5.3%
		%	57.8	52.8	48.3	48.3	53.7	52.8	48.5	57.1	63.9	63.3	56.2	64.4	64.1	63.6	70.8	62.5	9.7	18.4%
	**4**	Number	1381	97	98	92	87	90	113	83	87	92	93	105	86	96	79	83	-14	-14.4%
		%	58.3	59.1	57.3	54.1	48.3	51.4	59.2	54.6	58.0	61.3	58.5	69.1	61.0	67.6	60.3	59.3	0.1	0.2%
	**5 = least** **deprived**	Number	1342	95	78	81	98	83	90	99	88	89	89	93	115	95	77	72	-23	-24.2%
		%	55.9	54.6	51.0	48.2	49.2	48.0	51.7	54.7	52.4	62.2	58.9	59.2	68.0	68.3	60.2	59.0	4.4	8.1%
Term	**1 = most** **deprived**	Number	1009	56	68	70	80	102	85	64	88	71	63	60	52	46	56	48	-8	-14.3%
		%	38.2	41.2	42.8	39.5	38.5	48.8	42.7	38.3	48.4	40.6	34.2	33.1	29.2	28.8	32.7	31.2	-10.0	-24.3 %
	**2**	Number	986	56	78	81	73	96	78	76	74	71	63	49	39	47	55	50	-6	-10.7%
		%	40.7	37.8	46.4	46.6	41.2	50.5	46.7	46.9	45.4	42.3	39.9	36.6	26.5	29.9	33.7	34.2	-3.6	-9.5%
	**3**	Number	974	68	77	88	75	68	88	69	61	51	78	57	52	52	42	48	-20	-29.4%
		%	42.1	47.2	51.0	51.2	46.3	47.2	51.5	42.9	36.1	36.7	43.8	35.6	35.9	36.4	29.2	37.5	-9.7	-20.6%
	**4**	Number	987	67	73	78	93	85	78	69	63	58	66	47	55	46	52	57	-10	-14.9%
		%	41.7	40.9	42.7	45.9	51.7	48.6	40.8	45.4	42.0	38.7	41.5	30.9	39.0	32.4	39.7	40.7	-0.1	-0.3%
	**5 = least** **deprived**	Number	1057	79	75	87	101	90	84	82	80	54	62	64	54	44	51	50	-29	-36.7%
		%	44.1	45.4	49.0	51.8	50.8	52.0	48.3	45.3	47.6	37.8	41.1	40.8	32.0	31.7	39.8	41.0	-4.4	-9.7%

% based on births with known gestation and maternal SIMD quintile 2760 live singleton births had known gestation but unknown maternal SIMD quintile 58 live multiple births had known gestation but unknown maternal SIMD quintile Results not shown for post-term multiple births due to small numbers

**Table 10.** Preterm births to women in the most and least deprived categories, Scotland 2005–2019. (a) Percentage of live singleton births to women in the most (SIMD quintile 1) and least (SIMD quintile 5) deprived categories that were preterm, and absolute and relative difference between most and least deprived categories, Scotland 2005–2019

Singleton births Maternal SIMD quintile	Year														
	2005	2006	2007	2008	2009	2010	2011	2012	2013	2014	2015	2016	2017	2018	2019
**1 = most deprived** **preterm**	8.0	7.9	7.6	7.8	7.3	6.8	7.0	7.3	6.9	7.5	8.0	8.2	8.3	9.0	9.0
**5 = least deprived** **preterm**	5.1	4.9	5.0	5.2	5.2	4.4	5.1	4.6	4.7	5.2	5.8	5.0	5.4	5.0	5.4
**Absolute ** **difference 1–5**	2.9	3.0	2.6	2.7	2.1	2.5	1.9	2.7	2.2	2.4	2.2	3.2	2.9	4.0	3.6
**Ratio 1:5**	1.6	1.6	1.5	1.5	1.4	1.6	1.4	1.6	1.5	1.5	1.4	1.6	1.5	1.8	1.7

**Table:** (b) Percentage of live multiple births to women in the most (SIMD quintile 1) and least (SIMD quintile 5) deprived categories that were preterm, and absolute and relative difference between most and least deprived categories, Scotland 2005–2019

Multiple births Maternal SIMD quintile	Year														
	2005	2006	2007	2008	2009	2010	2011	2012	2013	2014	2015	2016	2017	2018	2019
**1 = most deprived** **preterm**	58.8	57.2	60.5	61.5	51.2	57.3	61.7	51.6	59.4	65.8	66.9	70.8	71.3	67.3	68.8
**5 = least deprived** **preterm**	54.6	51.0	48.2	49.2	48.0	51.7	54.7	52.4	62.2	58.9	59.2	68.0	68.3	60.2	59.0
**Absolute ** **difference 1–5**	4.2	6.3	12.2	12.3	3.2	5.6	7.0	-0.7	-2.8	6.8	7.6	2.7	2.9	7.1	9.8
**Ratio 1:5**	1.1	1.1	1.3	1.2	1.1	1.1	1.1	1.0	1.0	1.1	1.1	1.0	1.0	1.1	1.2

Over the whole study period, a reverse deprivation gradient in risk of post-term birth was evident. The percentage of singleton births that were post-term increased from 1.9% (3,913 / 204,992) amongst births to women in the most deprived quintile to 2.9% (3,800 / 131,830) amongst births to women in the least deprived quintile ( Table 9). The percentage of singleton births that were post-term decreased in all maternal deprivation categories between 2009 and 2019 ( Figure 4, Table 9).

### Multiple births

Of the 816,058 live births in Scotland between 2005 and 2019, 12,216 (1.5%) were multiple births (i.e., a birth following a multiple pregnancy resulting in one or more live baby), of which 12,198 (99.9%) had known gestational age at birth. The annual number of multiple births in Scotland initially increased from 769 in 2005 to 929 in 2008, then gradually declined to 696 in 2019. The percentage of all live births that were multiple was broadly static over our study period ( Table 1).

Over our study period, 7,158 (58.7%) multiple births were preterm, with 5,965 (48.9%) moderate to late preterm; 884 (7.2%) very preterm; and 309 (2.5%) extremely preterm. The percentage of all multiple births that were post-term was extremely low (<0.1%, 2 / 12,198). Of all multiple preterm births, 3,073 (42.9%) were spontaneous and 4,080 (57.0%) were provider-initiated (with 5 having unknown onset of labour) ( Table 1, Table 4). It is evident therefore that, for multiple pregnancies compared to singletons, the percentage of all births that are preterm is much higher; the percentage of preterm births that are at the lowest gestations is higher; and the percentage of preterm births that are spontaneous is lower (and conversely the percentage that are provider-initiated is higher).

**Figure 5.**
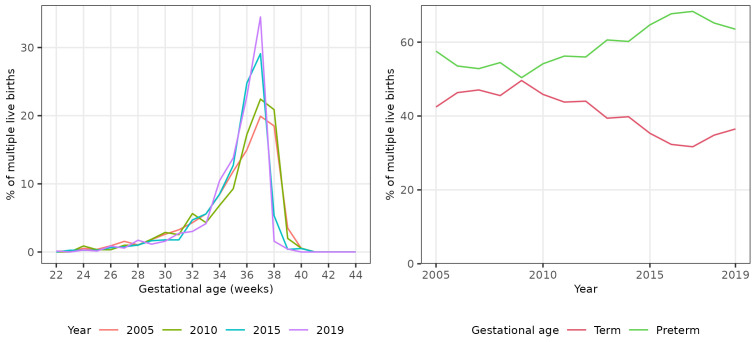
Trends in gestational age at birth, multiple live births, Scotland. **Figure 5a** shows the distribution of gestational age at birth for live multiple births in Scotland in 2005, 2010, 2015 and 2019. **Figure 5b** shows trends in the percentage of all live multiple births that were preterm and term in Scotland between 2005 and 2019.

**Figure 6.**
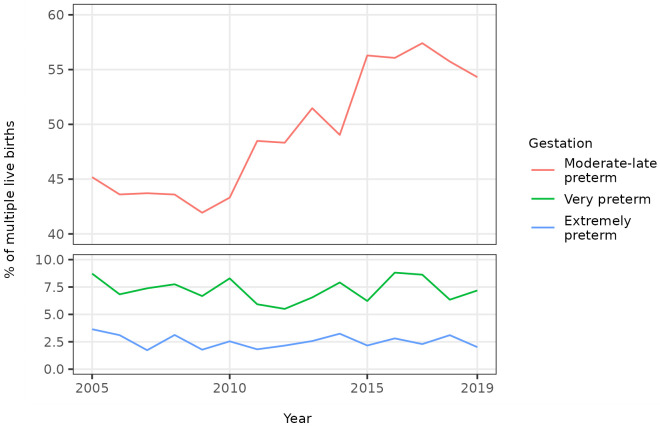
Trends in categories of preterm birth, multiple live births, Scotland. Figure 6 shows trends in the percentage of all live multiple births in Scotland between 2005 and 2019 that were moderate to late, very, and extremely preterm.

**Figure 7.**
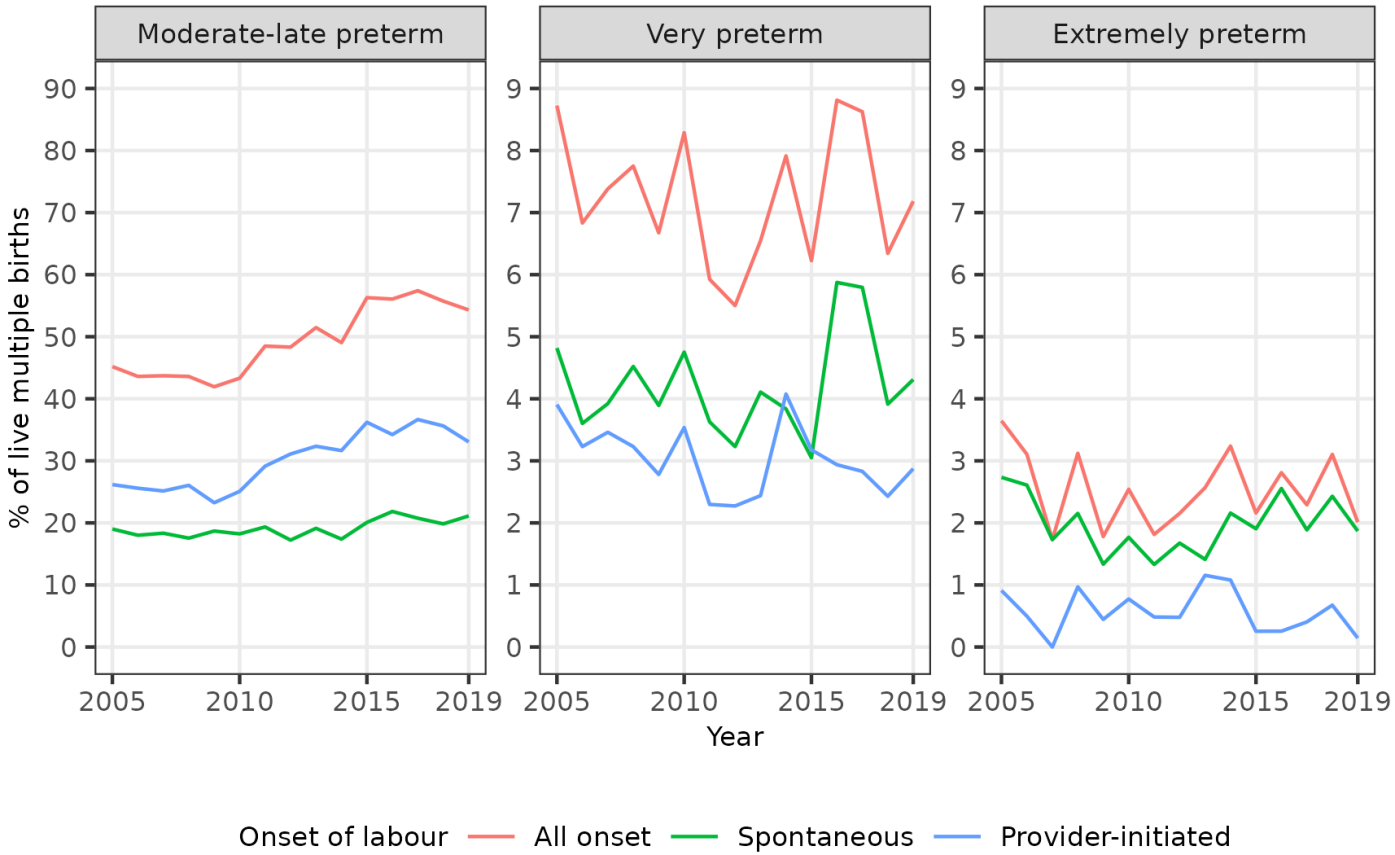
Trends in categories of preterm birth by onset of labour, multiple live births, Scotland. Figure 7 shows trends in the percentage of all live multiple births in Scotland between 2005 and 2019 that were moderate to late, very, and extremely preterm, by onset of labour (spontaneous or provider-initiated).

The percentage of multiple births that were preterm showed a similar trend over time as that noted for singletons, initially decreasing to 2009 then increasing to 2019 ( Figure 5). The increase was mainly driven by an increase in provider-initiated, moderate to late preterm multiple births ( Figure 6, Figure 7).

**Figure 8.**
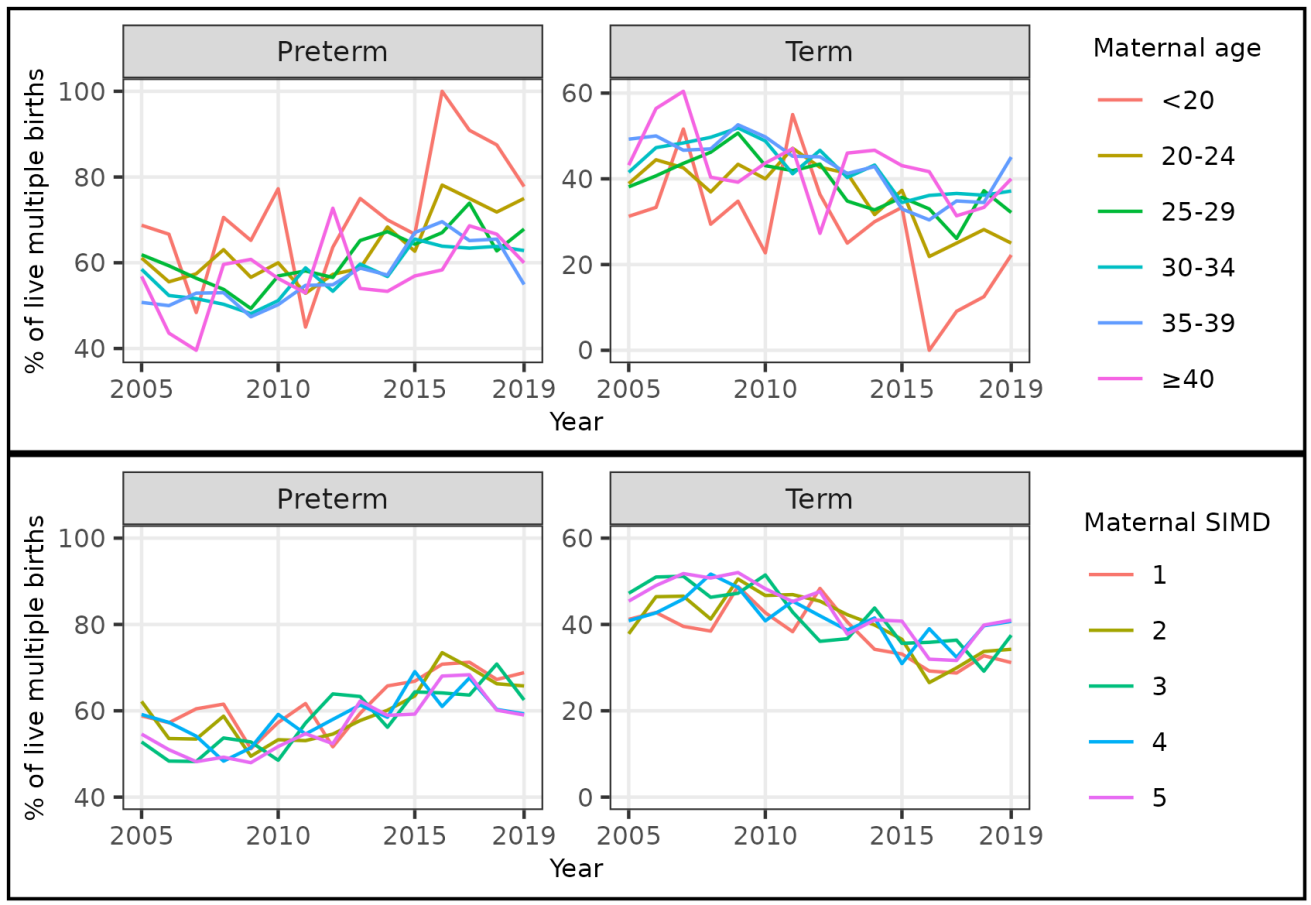
Trends in gestational age at birth by maternal age and deprivation, multiple live births, Scotland. **Figure 8a** shows trends in the percentage of all live multiple births in Scotland between 2005 and 2019 that were preterm and term by maternal age category **Figure 8b** shows trends in the percentage of all live multiple births in Scotland between 2005 and 2019 that were preterm and term by maternal Scottish Index of Multiple Deprivation (SIMD) quintile.

Over our study period, the percentage of multiple births that were preterm was highest for women in the youngest (but not oldest) age category and the most deprived category. However, as for singletons, the increase over time in the percentage of multiple births that were preterm was seen across all age and deprivation categories ( Figure 8, Table 5– Table 10).

## Discussion

### Summary of results

In this population-based study, we show that the percentage of live singleton births in Scotland that were preterm declined from 2005 to 2010, then increased to 2019. Although the total number of singleton births per year has declined over our study period, there was a higher number of singleton preterm births in 2019 than 2005. The percentage of singleton births that were spontaneous moderate to late, very, and extremely preterm all increased over time. By contrast, whilst the percentage of singleton births that were provider-initiated moderate to late preterm also increased over time, the percentage that were provider-initiated very or extremely preterm decreased over our study period. The percentage of singleton births that were preterm increased across all maternal age and deprivation categories, with increases greatest in groups at highest baseline risk (women in the youngest and oldest age categories, and the most deprived category). The percentage of live singleton births that were post-term showed a contrasting trend, increasing from 2005 to 2009, then decreasing to 2019.

For multiple births compared to singletons, the percentage of births that were preterm was much higher; the percentage of preterm births that were at the lowest gestations was higher; and the percentage of preterm births that were provider-initiated was higher. Despite these differences, the trend over time in the percentage of multiples births that were preterm was similar to that for singletons, decreasing from 2005 to 2009, then increasing to 2019. Most of the increase was due to an increase in the percentage of multiple births that were provider-initiated at moderate to late preterm. The percentage of multiple births that were post-term was extremely low.

### Strengths and limitations

In this population-based study, we examined trends in the percentage of live singleton and, separately, multiple births that were preterm, term, and post-term. For preterm births, we examined differences by category of preterm birth and onset of labour, allowing a nuanced understanding of trends. We explored trends over time by the key demographic variables of maternal age and deprivation category, allowing insight into inequalities. We draw on high quality, administrative data, with a high level of completeness and accuracy for key variables. Women booking for antenatal care in Scotland are routinely offered an ultrasound scan to inform pregnancy dating ^32^, and this has been the case since the 1990s ^33^. As 96% of pregnancies book at <15 weeks gestation in Scotland ^34^, we can be confident that gestational age was reliably and consistently measured over time using first trimester ultrasound ^35^, so changes in data quality are unlikely to explain observed trends in gestation at birth. The main limitations of this analysis were lack of data on additional important risk factors for gestational age at birth (e.g., weight, tobacco exposure, and pre-existing morbidities) and the lack of individual level data preventing multivariate analysis. We intended to examine trends by maternal ethnicity, but in a deviation from our study protocol were unable to do so due to high levels of missing data.

### Comparison with existing literature

This study builds on a previous analysis of trends in gestational age at live singleton birth in Scotland ^23^. The previous study reported a 42.2% and 10.7% relative increase in the provider-initiated and spontaneous preterm birth rate respectively from 1980 to 2004. Comparison with our findings of a 6.1% and 12.6% relative increase in the provider-initiated and spontaneous preterm birth rate from 2005 to 2019 suggests that the rate of increase in provider-initiated preterm birth has been slower since 2005, whilst the rate of increase in spontaneous preterm birth has been similar or higher. Our findings are in keeping with a previous analysis describing increasing preterm birth rates in high income countries prior to 2014 ^7^, but show that more recent Scottish trends diverge from the downward trend observed in other European countries immediately pre-pandemic ^19^. In describing recent long-term trends in post-term birth rates this paper addresses a gap in current literature.

### Interpretation

The observed increase over time in spontaneous preterm birth across all levels of prematurity, particularly for singletons, is concerning. This may reflect changes over time in the demographic characteristics of women giving birth (i.e., an increasing proportion of mothers having risk factors for spontaneous preterm birth) and/or increasing absolute risk for women with similar characteristics. Our results suggest that both factors may be contributing. There was an upward shift in maternal age over our study period, with an increasing proportion of mothers in the oldest age category that is at highest risk for preterm birth. However, we also show that the absolute risk of preterm birth increased for women in all age categories over time. We also show that the absolute risk of preterm birth increased for women in all deprivation categories over time, with the highest increase seen for women in the most deprived category. The increase in spontaneous pre-term birth was seen from 2010 onwards. The United Kingdom, including Scotland, experienced an economic recession in 2008/09, and 2010 marked the start of a period of subsequent sustained fiscal austerity. Our finding of increased preterm birth rates in this context is consistent with that of other studies showing the detrimental impact of economic recession and austerity on maternal and child health outcomes ^36– 38^.

The observed increase over time in provider-initiated moderate to late preterm birth for both singletons and multiples may represent improving ascertainment of foetal or maternal risk, changes in the underlying level of risk, and/or increasing clinician willingness to intervene to expedite birth at these gestations. Stillbirth is a competing risk for preterm live birth, and it is reassuring to note that stillbirth rates decreased in relative terms by 32% in Scotland between 2005 and 2019 ^39^. By contrast, the observed decrease in provider-initiated preterm birth at lower gestations (very and extremely preterm) over time, particularly for singletons, likely reflects a trend in clinical practice away from expediting birth at these very low gestations wherever possible.

The observed decrease over time in post-term birth for singletons is in keeping with accumulating evidence of the risks associated with expectant management of post-term pregnancy ^3– 6^ and in this respect is reassuring. The reverse deprivation gradient in post-term birth raises the possibility of a social patterning of decision making on management of post-term births, however. Further research is needed to confirm and understand underlying reasons for this.

## Conclusion

The percentage of all births in Scotland that were spontaneous preterm births increased from 2010 to 2019. Over the same time period, the percentage of births that were provider-initiated moderate to late preterm births also increased, but provider-initiated births at lower gestations decreased. The percentage of births that were post-term decreased.

Given the impact of gestational age at birth on both short and long-term health outcomes, and on health inequalities, ongoing monitoring of trends is important to identify changes during the pandemic period, the need for prevention, and effectiveness of preventive action. Analyses of individual level data quantifying the contribution of preventable risk factors to preterm birth, and incorporating the competing risk of stillbirth, are required.

## Ethics and consent

All analyses were undertaken within Public Health Scotland on anonymised, aggregate data following established organisational procedures. PHS has statutory authority to receive summary data returns on care provided by local NHS services and analyse the resulting national health datasets to support population health improvement. Local NHS services do not, therefore, ask patients for explicit consent before returning the agreed national data returns to PHS. PHS provides information for patients and the general public on how data is used, and how they can raise questions or concerns about that: for examples, see the PHS privacy notice and information provided through NHS Inform ( Health rights in Scotland | NHS inform). Analyses based on secondary analysis of aggregate data do not require external governance approval from the Public Benefit and Privacy Panel for Health and Social Care (see their Guidance for applicants documents). PHS has very strict internal processes governing how personal health data is handled. These types of analyses on aggregate data also do not require NHS ethical approval ( hra-decisiontools.org.uk) or review by the PHS ethics committee. In consequence, no additional ethical or information governance approvals were required for this analysis.

## Data Availability

We have not deposited the data separately to the manuscript as this analysis was conducted on aggregated data which are provided in the tables within the paper. We are not able to share patient-level data, but aggregate data from SMR02 is available through the Scottish Health and Social Care Open Data Platform ^29^. Bespoke research extracts of pseudonymised, patient level data can be provided through the Scottish national safe haven facility, subject to relevant approvals ^30^. The approvals process and criteria is detailed at Research data Scotland https://www.researchdata.scot. Analysis code available from: https://github.com/Public-Health-Scotland/preterm_trends_public Archived analysis code at time of publication: https://zenodo.org/doi/10.5281/zenodo.10952664 ^25^. License: Data are available under the terms of the Creative Commons Attribution 4.0 International license (CC-BY 4.0).
