# Supplementary material for: Trends in gestational age at live birth in Scotland from 2005 to 2019: a population-based study
Source: Wellcome Open Res. 2024 Oct 9;9:254. Originally published 2024 May 15. [Version 2] doi: 10.12688/wellcomeopenres.20916.2 (PMC11530745; doi:10.12688/wellcomeopenres.20916.2)
Supplement: Peer review history

# Reviewer report: Reviewer response for version 2

**DOI:** 10.21956/wellcomeopenres.25521.r106876

**Published:** 2024-11-12

**Authors:** Rosie Cornish

**Affiliations:** 1 University of Bristol, Bristol, England, UK

This manuscript describes trends in the prevalence of preterm births - overall and separately for spontaneous and provider-initiated preterm births - in Scotland between 2005 and 2019. The manuscript is well written and has been amended in response to previous reviewer comments. I only have one minor comment:

 1. The authors discuss the lack of data on additional risk factors for preterm birth as a limitation. One factor that they do not mention is parity. Given that the birth rate in the UK is decreasing, the proportion of live births among previously nulliparous women must be increasing. As this is a key factor associated with preterm birth, I think it would be worth specifically mentioning it in the manuscript.

Is the work clearly and accurately presented and does it cite the current literature?

Yes

If applicable, is the statistical analysis and its interpretation appropriate?

Yes

Are all the source data underlying the results available to ensure full reproducibility?

Yes

Is the study design appropriate and is the work technically sound?

Yes

Are the conclusions drawn adequately supported by the results?

Yes

Are sufficient details of methods and analysis provided to allow replication by others?

Yes

Reviewer Expertise:

epidemiology

I confirm that I have read this submission and believe that I have an appropriate level of expertise to confirm that it is of an acceptable scientific standard.

# Reviewer report: Reviewer response for version 2

**DOI:** 10.21956/wellcomeopenres.25521.r108030

**Published:** 2024-11-02

**Authors:** Laura Rachel Goodfellow

**Affiliations:** 1 University of Liverpool, Liverpool, UK

Thank you very much for inviting me to review this paper.

 This study uses national routinely collected data in Scotland to study trends in gestational age at birth from 2005 to 2019. The analysis is supplemented by stratification by maternal age and socioeconomic status.

 The paper is well written and clear. I think the discussion and conclusions are appropriate for the analysis and congratulate the authors on an interesting and informative paper.

**I only have two minor comments:**

1. Within figure 1a I’m struggling to follow the whole of the red line. I wonder whether it is overlayed with another line and so become not visible? I wonder whether it would be possible to implement colour blind friendly colours within your revision so that the figures are more accessible? It would also be a bit clearer if figures 1a and 1b and 5a and 5b had different coloured lines as I initially thought there was a relationship between the colours in the two adjacent graphs (as there are for your other adjacent graphs).
2. You discuss on page 24 the competing risks of stillbirth and (provider initiated) preterm birth. The outcome most important to parents is a live and healthy baby. It would have been fabulous within the current data to provide trends in stillbirth rates alongside the trends in preterm birth rates. This is obviously much more data and analysis and not appropriate at this stage of the study, but I wonder whether there was a reason why this wasn’t done that could be addressed within the limitations? The overall ‘bad news’ message of the paper (increase in preterm birth and increase in inequalities)- therefore we need to do something to address this- is somewhat diluted by the potential that at least some of the preterm births were a good thing to prevent stillbirth. It would be helpful for the reader to understand why stillbirths were excluded to understand whether further interrogation of this relationship is possible.

Is the work clearly and accurately presented and does it cite the current literature?

Yes

If applicable, is the statistical analysis and its interpretation appropriate?

Yes

Are all the source data underlying the results available to ensure full reproducibility?

Yes

Is the study design appropriate and is the work technically sound?

Yes

Are the conclusions drawn adequately supported by the results?

Yes

Are sufficient details of methods and analysis provided to allow replication by others?

Yes

Reviewer Expertise:

Preterm birth, preterm prelabour rupture of membranes (PPROM)

I confirm that I have read this submission and believe that I have an appropriate level of expertise to confirm that it is of an acceptable scientific standard.

# Reviewer report: Reviewer response for version 2

**DOI:** 10.21956/wellcomeopenres.25521.r104184

**Published:** 2024-11-01

**Authors:** Enny Cruz

**Affiliations:** 1 London School of Hygiene and Tropical Medicine, England, UK

I am happy to amend to approve the article.

Is the work clearly and accurately presented and does it cite the current literature?

Yes

If applicable, is the statistical analysis and its interpretation appropriate?

Partly

Are all the source data underlying the results available to ensure full reproducibility?

Yes

Is the study design appropriate and is the work technically sound?

Yes

Are the conclusions drawn adequately supported by the results?

Yes

Are sufficient details of methods and analysis provided to allow replication by others?

Yes

Reviewer Expertise:

Epidemiology

I confirm that I have read this submission and believe that I have an appropriate level of expertise to confirm that it is of an acceptable scientific standard.

# Reviewer report: Reviewer response for version 1

**DOI:** 10.21956/wellcomeopenres.23142.r92074

**Published:** 2024-08-22

**Authors:** Enny Cruz

**Affiliations:** 1 London School of Hygiene and Tropical Medicine, England, UK

This is a comprehensive descriptive analysis of the time trends in preterm birth rates in Scotland. The stratification by singleton and multiple births, as well as by segregation index, is particularly valuable.

**However, I have a few questions regarding the methods employed:**

1. Why did the authors choose logistic regression instead of linear regression for the statistical analyses? Additionally, how did they account for autocorrelation in the time series data?
2. Regarding the reported “absolute increase of 0.7% and a relative increase of 10.3%,” could you clarify what is meant by "absolute increase" and relative to what?
3. Phrases such as “decreased until 2010 before increasing until 2019” or “broadly static over the study period” suggest trends. Did you conduct any statistical tests for time trends to support these statements?
4. The text notes a shift in the distribution of maternal age at birth over the study period, with the percentage of singleton births to women aged <20 years decreasing from 7.9% (4,106 / 51,665) in 2005 to 3.0% (1,417 / 47,507) in 2019, and an increase in births to women aged ≥40 years from 3.2% (1,635 / 51,665) to 4.1% (1,925 / 47,507) in the same period (Table 5). Were any statistical tests performed to evaluate these changes? I also recommend merging and summarizing the subsequent paragraph for conciseness.

Moreover, why were post-term analyses by the onset of labor not included? 

 Finally, why were odds ratios (ORs) presented for only one part of the study, excluding multiple births? Neither the text nor the tables seem to provide this information.

 During a brief search, I found the following reference, which seems relevant for comparison with your findings: “Variation in rates of postterm birth in Europe: reality or artefact?” published in BJOG International Journal of Obstetrics and Gynaecology. Zeitlin *et.al (*2007 ^1^ )

Is the work clearly and accurately presented and does it cite the current literature?

Yes

If applicable, is the statistical analysis and its interpretation appropriate?

Partly

Are all the source data underlying the results available to ensure full reproducibility?

Yes

Is the study design appropriate and is the work technically sound?

Yes

Are the conclusions drawn adequately supported by the results?

Yes

Are sufficient details of methods and analysis provided to allow replication by others?

Yes

Reviewer Expertise:

Epidemiology

I confirm that I have read this submission and believe that I have an appropriate level of expertise to confirm that it is of an acceptable scientific standard, however I have significant reservations, as outlined above.

## References

[1] : Variation in rates of postterm birth in Europe: reality or artefact?. *BJOG* .2007;114(9) : 10.1111/j.1471-0528.2007.01328.x 1097-103 10.1111/j.1471-0528.2007.01328.x 17617197

# Response

**Published:** 2024-09-26

**Authors:** Emily Moore

**Affiliations:** Public Health Scotland, Glasgow, UK

Dear Dr Cruz, 

 Many thanks for taking time to review this paper and provide some important points for clarification. Please find a point-by-point response to each comment you made below.   Best wishes,  Dr Emily Moore  

**Comment: **Why did the authors choose logistic regression instead of linear regression for the statistical analyses? Additionally, how did they account for autocorrelation in the time series data? 

**Response: **Our study was focused on providing descriptive analyses of trends in the gestational distribution of births over time, and we used statistical modelling only to estimate a crude measure of the average annual change in the odds of our outcomes (i.e., preterm birth, term birth, post-term birth) over the study period.   When developing our protocol, we carefully considered different options for generating an estimate of the average annual change in our outcomes, including using a linear or Poisson model. We opted to take a relatively simple approach, given that we were only aiming to provide a crude estimate of the change over time and were not attempting to account for more complex temporal patterns or adjust for potential covariates. In our logistic regression model, each birth was considered as having the outcome or not (e.g., preterm versus not preterm) and calendar year was then included as a continuous exposure allowing us to assess the average increase in the odds of preterm birth with each increase in year.    

**Comment:** Regarding the reported “absolute increase of 0.7% and a relative increase of 10.3%,” could you clarify what is meant by "absolute increase" and relative to what? 

**Response: **We provided the following text in the Methods to describe the absolute and relative difference: *“We calculated the absolute and relative difference in the percentage of births that were preterm, term, and post-term in 2019 compared to 2005.  The absolute difference was calculated as the percentage in 2019 minus the percentage in 2005.  The relative difference was calculated by dividing the absolute difference by the baseline percentage in 2005 and multiplying by 100.”   * We have revised the text in the Results so it mirrors exactly the terms that we have described in the Methods: *“The percentage of all singleton births that were preterm increased from 6.5% in 2005 to 7.2% in 2019, giving an absolute difference of 0.7% (2019-2005) and a relative difference of 10.3% ((2019-2005)/2005x100). *    

**Comment**: Phrases such as “decreased until 2010 before increasing until 2019” or “broadly static over the study period” suggest trends. Did you conduct any statistical tests for time trends to support these statements? 

**Response**: These statements were based on visual inspection of our tabulated results and associated plots.  As noted above, this study mainly involved descriptive analyses. The only formal statistical testing we undertook was the logistic regression used to estimate the average annual change in the odds of the outcomes of interest.  We reflected carefully on the balance between descriptive analyses and statistical testing when developing our study protocol. We felt that a focus on descriptive analyses, using data visualisation to describe observed trends in the outcomes of interest was appropriate. In the context of a national, population-based analysis such as this, statistical testing will often give ‘significant’ results which may be of limited clinical relevance and difficult to interpret.  We have, however, been careful to ensure that detailed information on the numbers underlying all our results are available in the tables, which allows other researchers to do further exploration should they wish.   

**Comment: **The text notes a shift in the distribution of maternal age at birth over the study period, with the percentage of singleton births to women aged <20 years decreasing from 7.9% (4,106 / 51,665) in 2005 to 3.0% (1,417 / 47,507) in 2019, and an increase in births to women aged ≥40 years from 3.2% (1,635 / 51,665) to 4.1% (1,925 / 47,507) in the same period (Table 5). Were any statistical tests performed to evaluate these changes? I also recommend merging and summarizing the subsequent paragraph for conciseness. **Response:** As above, we did not conduct statistical testing on trends in the distribution of maternal age over time. This was not the focus of our study (we were interested in trends in the gestational distribution of births to women in different age groups), however we included it as important contextual information to support interpretation. For interested readers we have provided the numbers that would enable statistical testing of trends in the distribution of maternal age over time if they wanted to explore this further.  To make the results easy for readers to follow, we have tried to keep a consistent structure where possible in our manuscript. When looking at trends by both maternal age and deprivation, for example, we have structed the results as follows: 

- Paragraph one: description of distribution of all births by maternal age or deprivation 

- Paragraph two: description of the percentage of births that were preterm by maternal age or deprivation, over the total study period and the changes over time 

- Paragraph three: description of the percentage of births that were post-term by maternal age or deprivation, over the total study period and the changes over time 

We would therefore prefer to retain the current structure, and not combine the two paragraphs as suggested by the reviewer.    

**Comment:** Moreover, why were post-term analyses by the onset of labor not included? **Response: **As noted in the second paragraph of the Methods; Data source and quality section, Public Health Scotland (PHS) only applies the algorithm classifying mode of onset of labour to preterm births. We therefore did not have this data available for term or post-term births.  The reason that PHS only classifies preterm births reflects the specific clinical importance of distinguishing spontaneous and provider-initiated births in the preterm group to inform prevention. Spontaneous preterm birth is a pathological event, resulting from a range of possible underlying mechanisms, and trends are likely to directly reflect underlying trends in maternal and fetal health. Provider-initiated preterm birth is also likely to reflect concerns about the health of the mother or baby, but thresholds for initiating preterm delivery may vary over time hence trends are likely to reflect trends in both maternal and fetal health and clinical practice.  By contrast, the proportion of births that are post-term (≥42 weeks gestation) is likely to mainly reflect clinical practice, specifically the use of induction of labour towards the end of the term period which influences the proportion of pregnancies that progress into the post-term period. Reflecting current clinical guidelines e.g. from the National Institute for Health and Care Excellence (NICE), women with a post-term pregnancy are highly likely to be offered induction of labour, hence we would not expect to see the same variation in mode of onset of labour among post-term births as is seen for preterm births.   

**Comment:** Finally, why were odds ratios (ORs) presented for only one part of the study, excluding multiple births? Neither the text nor the tables seem to provide this information. **Response**:   All analyses were conducted for both singleton and multiple births (providing there was sufficient sample size). Table 3 presents the odds ratios for both singleton births (Table 3a) and multiple births (Table 3b); however, we appreciate that these need to be clearly signposted when reporting results for multiple births in the text. We have therefore added the following sentence to the “Multiple Births” section of the Results: *“The unadjusted odds of a multiple birth being preterm increased on average by 5% per year between 2005 and 2019 (OR 1.05, 95% CI 1.04 - 1.06) (Table 3).” *  

**Comment: **During a brief search, I found the following reference, which seems relevant for comparison with your findings: “Variation in rates of postterm birth in Europe: reality or artefact?” published in BJOG International Journal of Obstetrics and Gynaecology. Zeitlin et.al (2007 1) 

**Response: **Many thanks for highlighting this reference. We had not previously referred to this study as there are more recent estimates of post-term rates that we reference in our Introduction (reference: EuroPeristat. The European perinatal health report 2015-2019. EuroPeristat; 2020.  Available at https://www.europeristat.com/). We do agree with the reviewer that the data from Zeitlin et al’s study and the EuroPeristat publication could be compared to provide very crude estimates of changes over time (based only on two time points). We have removed the sentence in the Discussion that stated: “In describing recent long-term trends in post-term birth rates this paper addresses a gap in current literature”. We instead include the following sentence:  “Data reported separately for the year 2000 and for 2019 suggest that there was a decrease in post-term births over time in Scotland (19, 40), but our study provided annual measures of the percentage of births that were post-term allowing us to look in more detail at long-term trends.”   

# Reviewer report: Reviewer response for version 1

**DOI:** 10.21956/wellcomeopenres.23142.r90781

**Published:** 2024-08-15

**Authors:** Xianqiong Luo, Huazhang Miao

**Affiliations:** 1 Guangdong Women and Children Hospital, Guangzhou, China; 2 Guangdong Women and Children Hospital, Guangdong, China

This study used high quality, national, administrative data to examine trends in gestation at birth in Scotland, and have significant public health implications. The study design, statistical analysis and its interpretation is appropriate, the results and conclusions of this study are credible. All the source data underlying the results available to ensure full reproducibility.

Is the work clearly and accurately presented and does it cite the current literature?

Yes

If applicable, is the statistical analysis and its interpretation appropriate?

Yes

Are all the source data underlying the results available to ensure full reproducibility?

Yes

Is the study design appropriate and is the work technically sound?

Yes

Are the conclusions drawn adequately supported by the results?

Yes

Are sufficient details of methods and analysis provided to allow replication by others?

Yes

Reviewer Expertise:

Pediatric Endocrinology and Inherited Metabolic Diseases

We confirm that we have read this submission and believe that we have an appropriate level of expertise to confirm that it is of an acceptable scientific standard.
